# (Re)packing Equal Disks into Rectangle

**DOI:** 10.1007/s00454-024-00633-1

**Published:** 2024-03-12

**Authors:** Fedor V. Fomin, Petr A. Golovach, Tanmay Inamdar, Saket Saurabh, Meirav Zehavi

**Affiliations:** 1 University of Bergen, Bergen, Norway; 2 Indian Institute of Technology, Jodhpur, Jodhpur, India; 3 Institute of Mathematical Sciences, Chennai, India; 4 Ben-Guiron University, Beer-Sheva, Israel

**Keywords:** Computational geometry, Parameterized algorithms, Circle packing, Unit disks, 51E23: Spreads and packing problems, 68Q25: Analysis of algorithms and problem complexity, 68W40: Analysis of algorithms

## Abstract

The problem of packing of equal disks (or circles) into a rectangle is a fundamental geometric problem. (By a packing here we mean an arrangement of disks in a rectangle without overlapping.) We consider the following algorithmic generalization of the equal disk packing problem. In this problem, for a given packing of equal disks into a rectangle, the question is whether by changing positions of a small number of disks, we can allocate space for packing more disks. More formally, in the repacking problem, for a given set of *n* equal disks packed into a rectangle and integers *k* and *h*, we ask whether it is possible by changing positions of at most *h* disks to pack $n+k$ disks. Thus the problem of packing equal disks is the special case of our problem with $n=h=0$. While the computational complexity of packing equal disks into a rectangle remains open, we prove that the repacking problem is NP-hard already for $h=0$. Our main algorithmic contribution is an algorithm that solves the repacking problem in time $(h+k)^{\mathcal {O}(h+k)}\cdot |I|^{\mathcal {O}(1)}$, where |*I*| is the input size. That is, the problem is fixed-parameter tractable parameterized by *k* and *h*.

## Introduction

Packing of equal circles inside a rectangle or a square is one of the oldest packing problems. In addition to many common-life applications, like packing bottles or cans in a box [19], packings of circles have a variety of industrial applications, including circular cutting problems, communication networks, facility location, and dashboard layout [24]. We refer to the survey of Castillo et al. [7] for an interesting overview of industrial applications of circle packings.

The mathematical study of packing equal circles can be traced back to Kepler [23]. Packing of circles also poses exciting mathematical and algorithmic challenges. Significant efforts have been spent on variants of circle packing for several decades [26, 27, 30–32, 34, 35]. However, even in the simple setting of packing equal circles inside a square, the optimal bounds are known only for instances of up to tens of circles [33], and proving such optimal bounds remains a major problem in the area [10]. The computational complexity of packing of equal circles (NP-hardness or membership in NP) remains elusive. For packing circles with different radii, Demaine et al. claimed NP-hardness [12]. See also the work of Abrahamsen et al. [1] for a generic framework for establishing $\exists \mathbb {R}$-completeness for packing problems. There are also some recent results on packing circles (of possibly different radii) inside different containers achieving (near) optimal densities, based on the combined area of the circles—see [14, 33] and references therein.

**Fig. 1:**
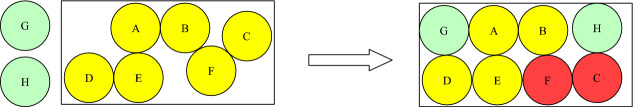
For a packing $\mathcal {P}$ of disks *A*–*F*, integers $h=2$, and $k=2$, the repacking $\mathcal {P}^*$ of $\mathcal {P}$ is obtained by relocating disks *C* and *F*, and by adding disks *G* and *H*

Our paper establishes several results on computational and parameterized complexity of a natural generalization of packing equal circles inside a rectangle. A remark in the terminology is in order. In the literature on packing, both terms, circles and disks, could be found. While the term circle is much more popular than disk, we decided to use disks for the following reason: in our results (especially the NP-hardness result), it is more convenient to operate with open disks. Thus all disks we consider are open and unit (that is, of radius one). Let us remind, that a family of disks forms a *packing* if they are pairwise nonintersecting.^1^ In our problem, we have a packing of disks in a rectangle, and the question is whether we can allocate some space for more disks by relocating a small amount of disks. More precisely, we consider the following problem. See Fig. 1 for an example.
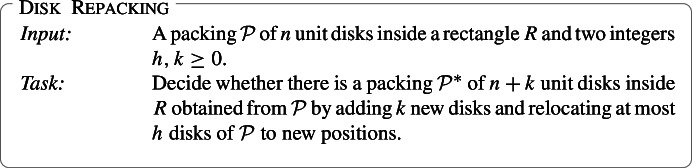

Thus when $n=0$, that is, initially there are no disks inside the rectangle, this is the classical problem of packing equal circles inside a rectangle.

*Related Work on Geometric Packing.* Packing problems have received significant attention from the viewpoint of approximation algorithms. For the sake of illustration, let us mention a few examples. In 2D Geometric Bin Packing, which is a variant of classical Bin Packing, the goal is to pack a given collection of rectangles into the minimum number of unit square bins. Typically, it is required that the rectangles be packed in an axis-parallel manner. There has been a long series of results on this problem, culminating in the currently known best approximation given by Bansal and Khan [5]. A related problem is that of 2D Strip Packing problem, where the task is to pack a given set of rectangles into an infinite strip of the given width, so as to minimize the height of packing. This problem has been studied from the context of approximation [20, 22] as well as parameterized [3] algorithms. Finally, we mention the Geometric Knapsack problem, which is also closely related to Geometric Bin Packing. In Geometric Knapsack, we are given a collection of rectangles, where each rectangle has an associated profit. The goal is to pack a subset of the given rectangles (without rotation) in an axis-aligned square knapsack, so as to maximize the total profit of the packed rectangles. Currently, the best approximation is given by Galvez et al. [17]. A detailed survey of the literature on the results of these problems is beyond the scope of this work—we direct an interested reader to the cited works and references therein and the survey paper of Christensen et al. [8]. However, we would like to highlight an important difficulty in Disk Repacking problem —which is the focus of this work—as compared to the aforementioned geometric packing problems, namely, that packing disks in a rectangle requires the use of intricate geometric arguments as compared to packing rectilinear objects (such as rectangles) in a rectilinear container (such as a unit square, or an infinite strip).

*Our Results.* We show that Disk Repacking problem-hard even if the parameter $h=0$—we call this special case of problem Disk Appending problem.

### Theorem 1.1

Disk Appending problem-hard when constrained to the instances $(R,\mathcal {P},k)$ where $R=[0,a]\times [0,b]$ for positive integers *a* and *b* and the centers of all disks in $\mathcal {P}$ have rational coordinates. Furthermore, the problem remains ${{\,\textrm{NP}\,}}$-hard when it is only allowed to add new disks to $\mathcal {P}$ with rational coordinates of their centers.

From the positive side, we show that Disk Repacking problem when parameterized by *k* and *h*. As it is common in Computational Geometry, we assume the *real RAM* computational model, that is, we are working with real numbers and assume that basic operations on these numbers can be executed in unit time. We use |*I*| to denote the input size of an instance *I*.

### Theorem 1.2

The Disk Repacking problem is ${{\,\textrm{FPT}\,}}$ when parameterized by $k+h$. Specifically, it is solvable in time $(h+k)^{\mathcal {O}(h+k)}\cdot |I|^{\mathcal {O}(1)}$.

Theorem 1.2 also appears to be handy for approximating the maximum number of disks that can be added to a packing. In the optimization variant of Disk Repacking problem, called Max Disk Repacking problem, we are given a packing $\mathcal {P}$ of *n* disks in a rectangle *R* and an integer *h*, and the task is to maximize the number of new disks that can be added to the packing if we are allowed to relocate at most *h* disks of $\mathcal {P}$. By combining Theorem 1.2 with the approach of Hochbaum and Maass [21], we prove that the optimization variant of Disk Repacking problem the parameterized analog of EPTAS for the parameterization by *h*. More precisely, we prove the following theorem.

### Theorem 1.3

For any $0< \varepsilon < 1$, there exists an algorithm that, given an instance $(\mathcal {P},R,h)$ of Max Disk Repacking problem, returns a packing $\mathcal {P}^*$ into *R* with at least $n + (1-\varepsilon ) \cdot \textsf{OPT}_h$ disks in time

$$\begin{aligned}\max \left\{ \left( \frac{h+1}{\varepsilon }\right) ^{\mathcal {O}(h/\varepsilon )}, \left( \frac{1}{\varepsilon }\right) ^{\mathcal {O}(1/\varepsilon ^2)}\right\} \cdot |I|^{\mathcal {O}(1)} \le \big (\frac{h+1}{\varepsilon }\big )^{\mathcal {O}(h/\varepsilon +1/\varepsilon ^2)} \cdot |I|^{\mathcal {O}(1)}, \end{aligned}$$

where $\textsf{OPT}_h$ is the maximum number of disks that can be added to the input packing if we can relocate at most *h* disks.

## Preliminaries

*Disks and rectangles.* For two points *A* and *B* on the plane, we use *AB* to denote the line segment with endpoints in *A* and *B*. The *distance* between $A=(x_1,y_1)$ and $B=(x_2,y_2)$ or the *length* of *AB*, is $|AB|=\Vert A-B\Vert _2=\sqrt{(x_1-x_2)^2+(y_1-y_2)^2}$. The *(open unit) disk* with a *center* $C=(c_1,c_2)$ on the plane is the set of points (*x*, *y*) satisfying the inequality $(x-c_1)^2+(y-c_2)^2<1$. Whenever we write “disk” we mean an open unit disk, unless explicitly specified otherwise. Throughout the paper, we assume that the input rectangle *R* is of the form $[0,a]\times [0,b]$ for some $a,b>0$.

*Parameterized Complexity.* We refer to the book of Cygan et al. [11] for an introduction to the area and undefined notions. A *parameterized problem* is a language $L\subseteq \Sigma ^*\times \mathbb {N}$, where $\Sigma ^*$ is a set of strings over a finite alphabet $\Sigma$. An input of a parameterized problem is a pair (*x*, *k*), where $x\in \Sigma ^*$ and $k\in \mathbb {N}$ is a *parameter*. A parameterized problem is *fixed-parameter tractable* (or ${{\,\textrm{FPT}\,}}$) if it can be solved in time $f(k)\cdot |x|^{\mathcal {O}(1)}$ for some computable function *f*.

*Systems of Polynomial Inequalities.* We use the following result from the book of Basu et al. [6]. We refer to the same book [6] for the background on terminology and the basic tools.

### Proposition 2.1

([6, Thm. 13.13]) Let *R* be a real closed field, and let $\mathcal {P} \subseteq R[X_1, \ldots , X_\ell ]$ be a finite set of *s* polynomials, each of degree at most *d*, and let

$$\begin{aligned}(\exists X_1) (\exists X_2) \ldots (\exists X_\ell ) F(X_1, X_2, \ldots , X_\ell )\end{aligned}$$

be a sentence, where $F(X_1, \ldots , X_\ell )$ is a quantifier-free boolean formula involving $\mathcal {P}$-atoms of type $P \odot 0$, where $\odot \in \{ =, \ne , >, < \}$, and *P* is a polynomial in $\mathcal {P}$. Then, there exists an algorithm to decide the truth of the sentence with complexity $s^{\ell +1} d^{\mathcal {O}(\ell )}$ in *D*, where *D* is the ring generated by the coefficients of the polynomials in $\mathcal {P}$.

Furthermore, a point $(X_1^*,\ldots ,X_\ell ^*)$ satisfying $F(X_1, \ldots , X_\ell )$ can be computed in the same time by Algorithm 13.2 (sampling algorithm) of [6] (see Theorem 13.11 of [6]). Note that because we are using the real RAM model in our algorithms, the basic operations on real numbers can be performed in unit time. Thus, the complexity of our algorithms is stated with respect to the natural parameters, i.e., the input size, as well as, additional parameters such as $h, k, \varepsilon$.

## Hardness of Disk Appendingproblem

In this section, we prove Theorem 1.1 on the hardness of Disk Appending problem. Recall, that Disk Appending problem the special case of Disk Repacking problem. We use the following auxiliary notation in this section.

We use standard graph-theoretic terminology and refer to the textbook of Diestel [13] for missing notions. We consider only finite undirected graphs. For a graph *G*, *V*(*G*) and *E*(*G*) are used to denote its vertex and edge sets, respectively. For a vertex $v\in V(G)$, we denote by $N_G(v)=\{u\in V(G)\mid uv\in E(G)\}$ the *neighborhood* of *v*, and $d_G(v)=|N_G(v)|$ is the *degree* of *v*. A graph is *cubic* if every vertex has degree three. A graph *G* is *planar* if it has a planar embedding, that is, it can be drawn on the plane without crossing edges. A *rectilinear* embedding is a planar embedding of *G* such that vertices are mapped to points with integer coordinates and each edge is mapped into a broken line (or a piecewise linear curve) consisting of an alternate sequence of horizontal and vertical line segments. The switches between horizontal and vertical lines are called *bends*. The *area* of an embedding is the minimal $(b_1-a_1)(b_2-a_2)$ such that all points of the embedding are in the rectangle $[a_1,b_1]\times [a_2,b_2]$.

We say that a point *X* is *properly inside* of a polygon *P* if it is inside *P* but *X* is not on the boundary; if we say that *X* is inside *P*, we allow it to be on the boundary. A disk is (*properly*) *inside* of a polygon *P* if every point of the disk is (properly) inside of *P*.

We restate the main theorem of the section.

### Theorem 3.1

Disk Appending problem-hard when constrained to the instances $(R,\mathcal {P},k)$ where $R=[0,a]\times [0,b]$ for positive integers *a* and *b* and the centers of all disks in $\mathcal {P}$ have rational coordinates. Furthermore, the problem remains ${{\,\textrm{NP}\,}}$-hard when it is only allowed to add new disks to $\mathcal {P}$ with rational coordinates of their centers.

**Proof of** **Theorem** 1.1: **Overview.** We reduce from the Independent Set problem. Let us recall that in this problem, for a given graph *G* and a positive integer *k*, the task is to decide whether *G* contains an independent set, that is a set of pairwise nonadjacent vertices, of size at least *k*. It is well-known that Independent Set is ${{\,\textrm{NP}\,}}$-complete on cubic planar graphs [18] (see also [28] for an explicit proof).

Before diving into the technical details, let us outline the main ideas of the reduction. Let *G* be a graph and assume that $\ell _e$ are positive integers given for all $e\in E(G)$. Suppose that $G'$ is obtained from *G* by subdividing each edge *e* exactly $2\ell _e$ times (the edge subdivision operation for $e=uv$ deletes *e* and creates a new vertex $w_e$ adjacent to both *u* and *v*). Then it can be shown that *G* has an independent set of size *k* if and only if $G'$ has an independent set of size $k+\sum _{e\in E(G)}\ell _e$. We exploit this observation. Given a rectilinear embedding of a cubic planar graph *G*, for each vertex of *G*, we create a *node* area formed by surrounding disks. We can place an additional disk in such an area and this encodes the inclusion of the corresponding vertex to an independent set. Then we join the areas created for vertices by *channels* corresponding to subdivided edges. Similarly to node areas, channels are formed by surrounding disks. Each channel contains an even number of positions where new disks can be placed, and these positions are divided into “odd” and “even” in such a way that we can put disks in either all odd or all even positions but no disks could be placed in adjacent even and odd positions. Thus node areas and channels are used to encode a graph, and then we fill the space around them by *filler* disks that prevent placing any new disk outside node areas and channels. Then placing new disks corresponds to the choice of an independent set in a subdivided graph. Further in this section, we give a formal proof of Theorem 1.1. To avoid unnecessary complications in the already technical proof, we allow algebraic number parameters in our reduction and then explain how we can get rid of these constraints.

**Proof of** **Theorem** 1.1: **Constructing channels and node areas.** Our construction of node areas and channels follows a rectilinear embedding of a planar graph and we use the fact that rectilinear embeddings can be constructed efficiently. In particular, the following theorem was shown by Liu, Morgana, and Simeone [25].

### Proposition 3.2

[25] Every *n*-vertex planar graph of maximum degree at most 4 admits a rectilinear embedding with at most 3 bends for every edge with the area $\mathcal {O}(n^2)$. Furthermore, such an embedding can be constructed in $\mathcal {O}(n)$ time.

**Fig. 2:**
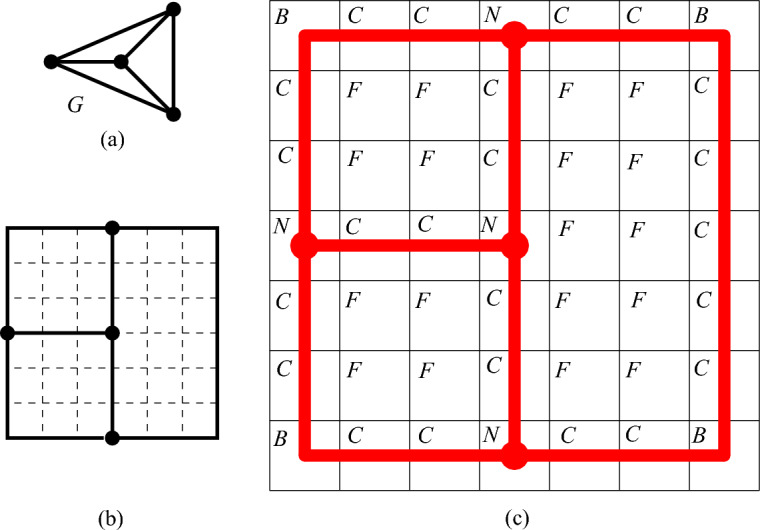
Encoding of the graph *G* shown in (**a**). A rectilinear embedding of *G* is shown in (**b**) and the encoding of *G* via tiles is shown in (**c**); the node areas and channels are shown in red, the node, channel, bend, and filler tiles are labeled by *N*, *C*, *B*, and *F*, respectively

We use Proposition 3.2 to construct the node areas and channels. Let *G* be an *n*-vertex cubic graph. We assume that we are given a rectilinear embedding of *G* with the properties guaranteed by Proposition 3.2. We also assume without loss of generality that the length of every segment of a broken line representing an edge is at least three. This can be achieved by replacing every vertex or bend point (*x*, *y*) of the embedding by the point (3*x*, 3*y*) and the corresponding adjustment of the segments in the broken lines. Notice that every segment in the embedding contains at least two integer points different from the endpoints of the segment. For each integer point of the rectangle containing the embedding, we construct a $2c\times 2c$ square tile, where *c* is a sufficiently big odd positive integer (the choice of *c* will be explained later), of one of the following four types: (i) node tile containing a node area, (ii) horizontal/vertical channel, (iii) bend channel tiles to form channels, and (iv) filler tile to fill forbidden areas. Then we use these tiles to encode a graph as shown in Fig. 2 sticking the tiles together following the embedding.

**Fig. 3:**
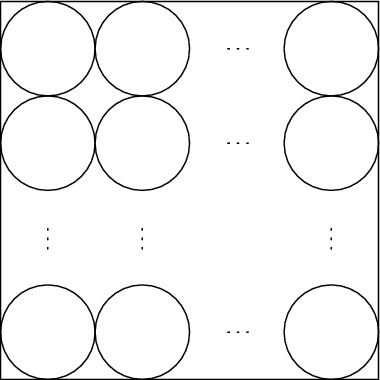
The filler tile

Now we describe these tiles. We start with the construction of the filler tile which is trivial—we simply fill a $2c\times 2c$ square by disks as shown in Fig. 3.

**Fig. 4:**
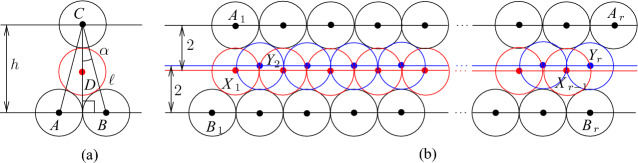
The basic channel of size *r*; the disks shown in red and blue are not parts of the channel—they show places where new disks can be inserted

Next, we deal with channel tiles. The construction of these tiles is more complicated. In particular, we need three kinds of such tiles because we have to adjust parities and join them together with other tiles. However, the basic idea is the same for all kinds. Consider four touching disks with centers *A*, *B*, *C*, and *D* shown in Fig. 4a. Note that $h=2+\sqrt{3}$, $\ell =|AC|=|BC|=2\sqrt{2+\sqrt{3}}$, and the angle $\alpha =\pi /12$. Then we can make the straightforward observation that, given disks with centers at *A*, *B*, and *C*, every disk with its center in the triangle *ABC* has its center at *D*. Then extending this, we can make the following observation about the configuration of disks shown in Fig. 4b. We call such a configuration of disks a *basic channel* of size *r*. When we say that a disk is placed or added, we mean that the disk should be disjoint with other disks. Also, we say that a disk is *inside* of a channel if its center is in $B_1A_1A_rB_r$.

### Observation 3.3

Given disks with centers at $A_1,\ldots ,A_r$ and $B_1, \ldots ,B_r$ as shown in Fig. 4b, any disk placed properly inside the quadrilateral $A_1B_1B_rA_r$ has its center at one of the points $X_1,\ldots ,X_{r-1}$ or $Y_2,\ldots ,Y_r$. Furthermore, if a disk with its center at $X_i$ ($Y_i$, respectively) is placed in the quadrilateral then no other disk can have its center at $Y_{i}$ or $Y_{i+1}$ ($X_{i-1}$ or $X_i$, respectively).

**Fig. 5:**
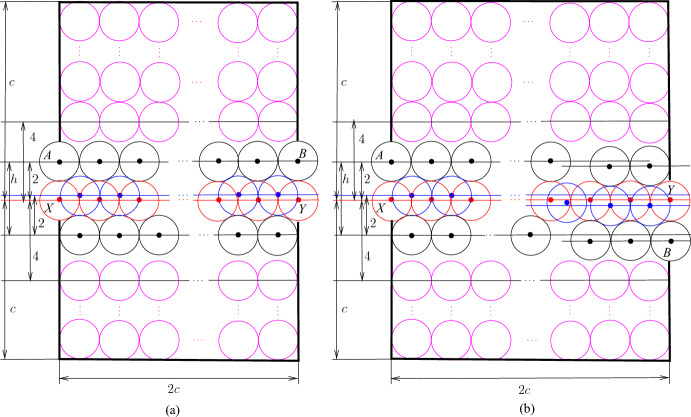
The straight channel tile (**a**) and the twisted channel tile (**b**). The disks shown in red and blue are not parts of the gadgets, the disks shown in magenta are used to fill space

We use basic channels to construct channel, bend, and node tiles. In particular, we construct the *straight* channel tile from the basic channel of size *c* by deleting the left bottom disk and filling the space outside the channel in the $2c\times 2c$ square by additional disks as shown in Fig. 5a. The disks with the centers at *A* and *B* are called *poles*. They are identified with poles of other tiles to join them together. We refer to the basic channel inside the tile as the *channel of the tile*.

**Fig. 6:**
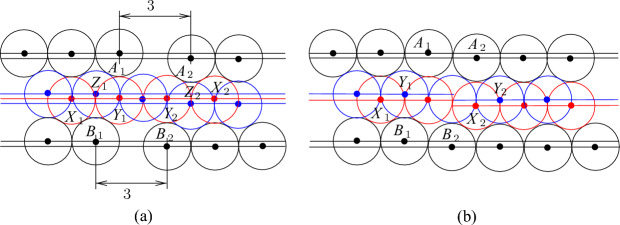
Twisting (**a**) and level adjustment (**b**)

However, we need some further configurations of disks because we have to adjust parities and distances in tiles, and also we have to join tiles with each other. In particular, to join channel tiles with other tiles, we have to twist basic channels in some of them as shown in Fig. 6a. Then we can make the following observation.

### Observation 3.4

Given disks with centers at $A_1,A_2$ and $B_1,B_2$ as shown in Fig. 6a, the following holds:

- if two disks with their centers at $X_1$ and $X_2$ are placed as shown in Fig. 6a then at most two disks with their centers inside $A_1B_1B_2A_2$ can be added, and if two disks are placed in $A_1B_1B_2A_2$ they have centers at $Y_1$ and $Y_2$, respectively,
- if two disks with their centers at $Z_1$ and $Z_2$ are placed as shown in Fig. 6a then it is possible to place one disk with its center inside $A_1B_1B_2A_2$ but at most one such a disk can be added.

### Proof

To see the first claim, notice that $Y_1$ and $Y_2$ compose a unique pair of points in the quadrilateral $A_1B_1B_2A_1$ such that $|Y_1Y_2|\ge 2$ and $|A_iY_j|,|B_iY_j|\ge 2$ for $i,j\in \{1,2\}$ (in fact, $|Y_1Y_2|=2$ and $|A_iY_j|,|B_iY_j|=2$). For the second claim, note that if there are two disks with their centers in $Z_1$ and $Z_2$, respectively, then for any disk with its center *Y* inside $A_1B_1B_2A_2$, it must hold that $|Z_iY|,|A_iY|,|B_iY|\ge 2$ for $i\in \{1,2\}$. Then we can only choose *Y* to be the middle point between $Y_1$ and $Y_2$ and place a disk having its center in *Y*. However, we cannot place two such disks because for any *Y* and $Y'$ in $A_1B_1B_2A_2$ at distance at least two from $A_1,A_2,B_1,B_2,Z_1,Z_2$, $|YY'|<2$. $\square$

We construct the *twisted* channel tile (see Fig. 5b) similarly to the straight channel tile—the difference is that we insert one twist using Observation 3.4. The crucial properties of straight and twisted channel tiles are given in the following lemma. We say that a point is *inside a tile* if it is inside of the $2c\times 2c$ square in the tile.

### Lemma 3.5

At most $c+1$ new disks having their centers in the (straight, twisted) channel tile can be added and it is possible to place $c+1$ disks. Moreover, the following holds:

- only disks inside channels can be added,
- if exactly $c+1$ disks are placed then two of them have their centers at *X* and *Y* (see Fig. 5),
- it is possible to place *c* disks that have no centers at *X* and *Y* but then they are completely inside the tile and it is impossible to place an additional disk having its center inside the tile.

### Proof

The claims for the straight channel tile immediately follow from Observation 3.3 and the construction of the tile. In particular, to see the last claim, notice that if there are no disks with their centers at *X* and *Y* then only disks colored blue in Fig. 5 can have their centers in the tile. For the twisted channel tile, we combine Observation 3.3 and Observation 3.4. $\square$

Our channel gadget (see Fig. 4) ensures that we have two options for the placements of disks within the channels. It is also important to ensure that the number of disks of each type that can be placed in a channel is exactly the same. However, if we construct a channel by joining straight and twisted channel tiles, we obtain that the number of disks colored red in Fig. 5 is bigger than the number of blue disks. To fix this, we add one special tile, called the *parity adjustment* channel tile, in each channel. To construct such a tile, we have to take into account that disks placed inside a basic channel may be on different levels (see the red and blue disks in Fig. 4b with their centers on the red and blue line, respectively). Hence, we need to adjust levels as shown in Fig. 6b. Then we observe the following.

### Observation 3.6

Suppose that we are given disks with centers at $A_1,A_2$ and $B_1,B_2$ as shown in Fig. 6b. Then if there are two disks with their centers at $X_1$ and $X_2$ ($Y_1$ and $Y_2$, respectively), at most one disjoint disk with its center at $A_1B_1B_2A_2$ can be added.

**Fig. 7:**
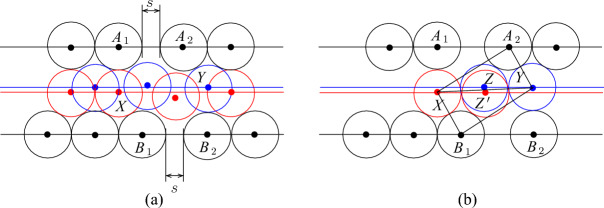
Gap insertion

To fix parity, we also have to adjust distances. For this, we observe that we can insert gaps of length $s<\sqrt{4\sqrt{3}-3}-1$ between disks in basic channels as shown in Fig. 7a.

### Observation 3.7

Given disks with centers at $A_1,A_2$ and $B_1,B_2$ as shown in Fig. 7a, at most one disk with its center inside the quadrilateral $A_1B_1B_2A_2$ can be added. Furthermore, if a disk has a center inside $A_1B_1B_2A_2$ then this disk intersects the disk with its center at *X* or the disk with its center at *Y*.

### Proof

The claim follows from the following geometrical observation illustrated in Fig. 7b. Suppose that the gap is exactly $\sqrt{4\sqrt{3}-3}-1$ and there are disks with centers at $A_1$, $A_2$, $B_1$, $B_2$, *X* and *Y*. Then any disk with its center inside $A_1B_1B_2A_2$ either has its center in the triangle $XYA_2$ or the triangle $XYB_1$. In the first case, the only possible center is *Z* and the disk with its center in *Z* touches the disks with centers *X*, *Y*, and $A_2$—the point *Z* is uniquely defined by this touching conditions. Similarly, if the center is in $XYB_1$ then this disk has its center in the unique point $Z'$ at distance exactly two from *X*, *Y*, and $B_1$. This implies that if $s<\sqrt{4\sqrt{3}-3}-1$ then no new disk can be inserted in $A_1B_1B_2A_2$. $\square$

**Fig. 8:**
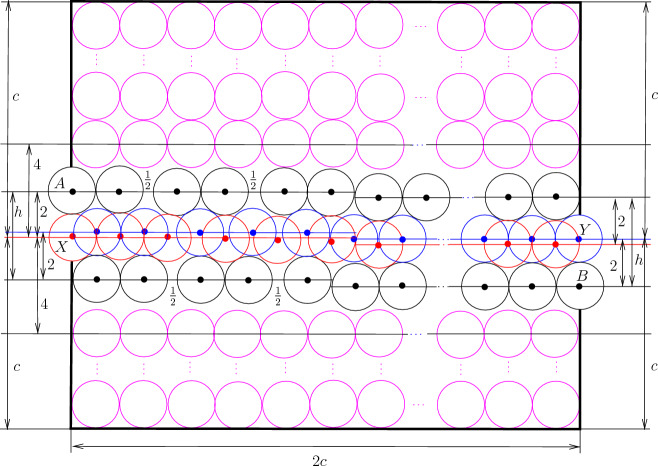
The parity adjustment channel tile. The disks shown in red and blue are not parts of the gadgets, the disks shown in magenta are used to fill space

Now we construct the *parity adjustment* channel tile from the basic channel of size $c-1$ by introducing two gaps of size $1/2<\sqrt{4\sqrt{3}-3}-1$ and one level adjustment as it is shown in Fig. 8. For the parity adjustment channel tile, we have the following properties.

### Lemma 3.8

At most *c* new disks having their centers in the parity adjustment channel tile can be added and it is possible to place *c* disks. Moreover, only disks inside the channel of the tile can be added, and if *c* disks are added then either one of them has its center at *X* (see Fig. 8) and it is impossible to add the disk having its center at *Y* or, symmetrically, one disk has its center at *Y* and the disk centered at *X* cannot be inserted.

### Proof

The proof is very similar to the proof of Lemma 3.5 and is obtained by combining Observations 3.3, 3.6, and 3.7. $\square$

**Fig. 9:**
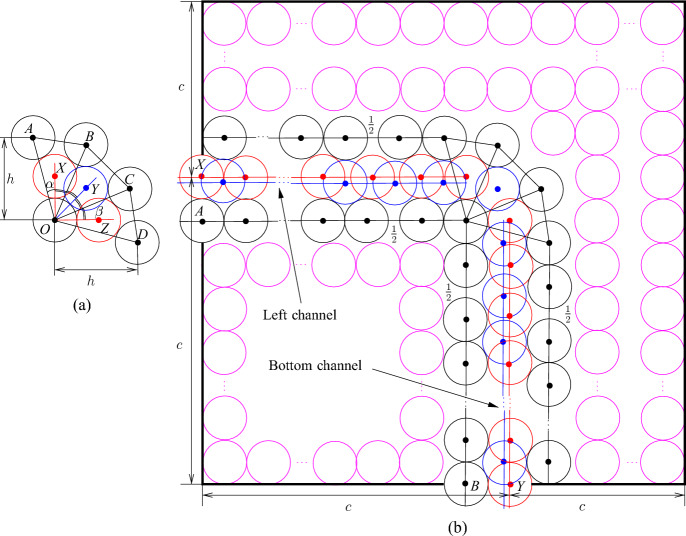
The bend tile. The disks shown in red and blue are not parts of the gadgets, the disks shown in magenta are used to fill space

We use basic channels and apply gap insertions to construct the bend tile. Additionally, we observe that we can “bend” basic channels (see Fig. 9a). Consider five touching disks with centers *A*, *B*, *C*, *D*, and *O* shown in Fig. 9a; $h=2+\sqrt{3}$, $|OA|=|OD|=\ell =2\sqrt{2+\sqrt{3}}$, $|OB|=|OC|=2\sqrt{2+\sqrt{2}}$, and the angles $\alpha =\pi /12$ and $\beta =\pi /8$. Then we can make the following observation.

### Observation 3.9

Given disks with their centers at *A*, *B*, *C*, *D* and *O*, only disks with centers at *X*, *Y* and *Z* can have their centers in *ABCDO*. Moreover, if there is a disk with its center at *X* or *Z* then the disk with its center at *Y* cannot be added, and if there is a disk with its center at *Y* then no disk having its center if *X* or *Z* can be added.

Observation 3.9 allows to construct the bend tile (see Fig. 9b). We use the configuration of disks from Fig. 9a and attach two basic channels called *left* and *bottom* channels, respectively. To adjust distances, we insert two gaps of size 1/2 into each channel. Then the remaining space if filled by disks. The disks with their centers at *A* and *B* are *poles* of the tile. For the bend tile, we have the following properties.

### Lemma 3.10

At most $c-1$ new disks having their centers in the bend tile can be added and it is possible to place $c-1$ disks. Moreover, the following holds:

- disks can be placed only inside the channel,
- if exactly $c-1$ disks are placed then two of them have their centers at *X* and *Y* (see Fig. 9a),
- it is possible to place $c-2$ disks that have no centers at *X* and *Y* but then they are completely inside the tile and it is impossible to place an additional disk having its center inside the tile.

### Proof

The proof immediately follows from Observations 3.3, 3.7, and 3.9. $\square$

**Fig. 10:**
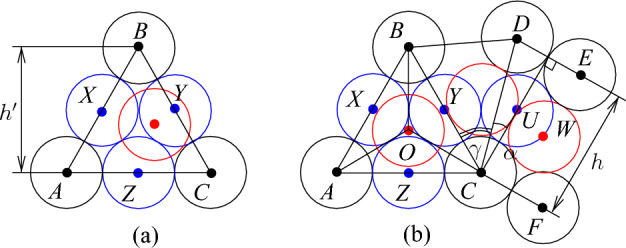
Node area (**a**) and the attachment of a basic channel to the node area

The construction of the node tile is based on the following geometric observations. Consider an equilateral triangle *ABC* with sides of length two as shown in Fig. 10a, $h'=2\sqrt{3}$. Suppose that there are disks with centers at *A*, *B*, and *C*. Then it is possible to place at most three disks with centers in the triangle *ABC*, and if exactly three disks are placed then they have their centers at *X*, *Y* and *Z* and touch each other. Furthermore, if a disk having its center properly inside *ABC* is placed then no other disk with its center inside the triangle can be added. We exploit this property and add a basic channel as shown in Fig. 10b. The point *O* is the center of *ABC*, that is, $|OA|=|OB|=|OC|$. Recall that $h=2+\sqrt{3}$ and $\alpha =\pi /12$. We set $\gamma =\pi /3-\pi /12=\pi /4$. This gives us the configuration of disks with the following properties summarized in the next observation.

### Observation 3.11

Given disks with centers at *A*, *B*, *C*, *D*, *E* and *F* as shown in Fig. 10b, the following is fulfilled:

- at most one disk with its center in *BCD* can be added,
- if there is a disk with its center either at *Y* or *U* then no other disk can have its center properly in *BCD*,
- if there are disks with their centers at *O* and *W* then a disk with its center in *BCD* can be added,
- if there is a disk having its center properly inside *ABC* then no other disk with its center inside *ABC* can be added.

### Proof

To see the first claim, notice that $|CB|=4$, $|BD|<4$, and $|CD|<4$. Then for any two points $P_1$ and $P_2$ in *BCD* such that $|P_iB|,|P_iC|,|P_iD|\ge 2$ for $i\in \{1,2\}$, we have that $|P_1P_2|<2$.

For the second claim, notice that if there is a disk with its center at *Y* then no other disk can have its center in *BCD* by the first claim. Suppose that there is a disk with its center at *U*. Notice that $|BU|<4$ and the disk centered at *Y* touches the disks with their centers at *B*, *C*, and *U*. This implies that no disk can have its center properly inside *BCD*.

The third claim follows from the observation that the disk that touches the disks with the centers at *C*, *D*, and *W* does not intersect the disk with its center at *O*.

The final claim immediately follows from the observation that $|AB|=|BC|=|AC|=4$ and the disks with the centers at *X*, *Y*, and *Z* touch each other and touch the disks with the centers at *A*, *B*, and *C* (see Fig. 10a). $\square$

**Fig. 11:**
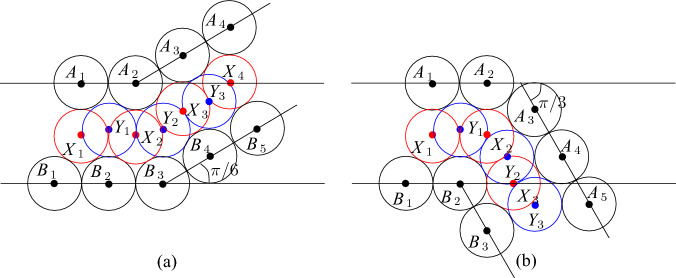
Bending of basic channels

We also use an easy observation that the basic channel construction allows us to bend them by $\pi /6$ or $\pi /3$ as shown in Fig. 11.

### Observation 3.12

Given disks with centers at $A_1,\ldots ,A_4$ and $B_1, \ldots ,B_5$ as shown in Fig. 11a, any disk with its center inside the quadrilaterals $A_1A_2B_3B_1$ or $A_2A_4B_5B_3$ has its center at one of the points $X_1,\ldots ,X_4$ or $Y_1,Y_2,Y_3$. Similarly, if disks with centers $A_1,\ldots ,A_5$ and $B_1, \ldots ,B_3$ are placed as shown in Fig. 11b then any disk with its center inside the quadrilaterals $A_1A_2B_2B_1$ or $A_3A_5B_3B_2$, or the triangle $A_2A_3B_2$ has its center at one of the points $X_1,X_2,X_3$ or $Y_1,Y_2,Y_3$. Furthermore, if a disk with its center at $X_i$ ($Y_i$, respectively) is placed then no other disk can have its center at $Y_{i-1}$ or $Y_{i}$ ($X_{i}$ or $X_{i+1}$, respectively).

**Fig. 12:**
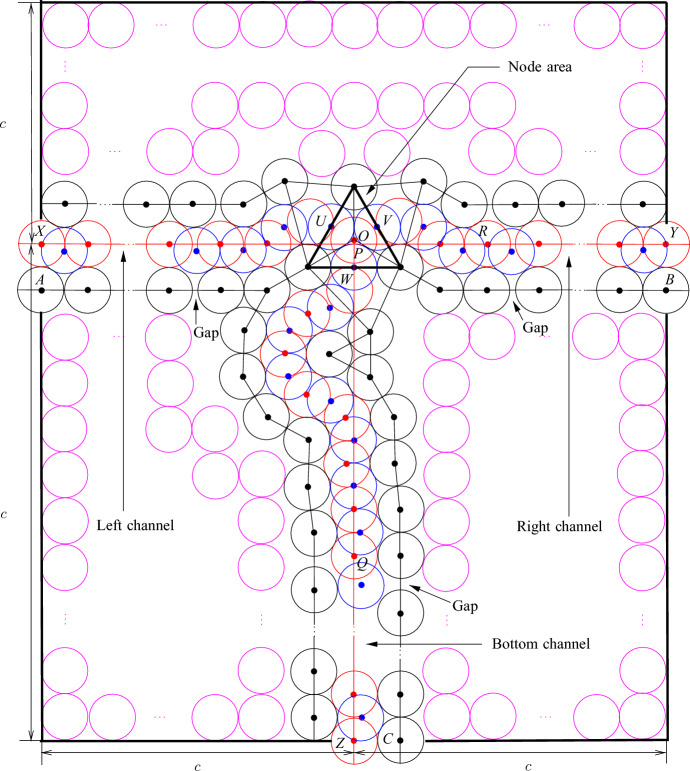
The node tile. The disks shown in red and blue are not parts of the gadgets, the disks shown in magenta are used to fill space. The point *P* is the center of the tile and the attachment of the left, right, and bottom channels is shown by black lines

Now we are ready to construct the node tile (see Fig. 12).

- We construct the node area formed by an equilateral triangle as shown in Fig. 10a.
- We attach three basic channels to the node area as shown in Fig. 10b; the channels are called *left*, *right* and *bottom*, respectively, as shown in Fig. 12.
- To construct the left (right, respectively) channel, we use a basic channel with $\pi /6$ bend as is it is shown in Fig. 11a. Notice that $|PR|=4+\sqrt{3}$, where *P* is the center of the tile and *R* is the point in the channel after the bend (see Fig. 12). To make the distances integer, we insert $2-\sqrt{3}$ gap in the basic channel (see Fig. 7a). Then we insert two gaps of length 1/2 to adjust distances.
- To construct the bottom channel, we bend the basic channel as shown in Fig. 11b to adjust the direction. Then we make the level adjustment (see Fig. 6b). Note that $|PQ|=2\sqrt{3}+11$, where *Q* is the point in the channel after the bending and level adjustment (see Fig. 12). We introduce $4-2\sqrt{3}$ gap and then we add 6 gaps of length 1/2 between parts of the basic channel to ensure that exactly the same number of disks could be placed in the bottom channel as in the left and right (this is due to the bend in the channel).
- The remaining space around the node area and the channel is filled by disks as shown in Fig. 12.

The disks with their centers at *A*, *B*, and *C* are called *poles* of the node tile.

The properties of the node tile are summarized in the next lemma.

### Lemma 3.13

At most $(c-1)/2$ disks can be placed inside each channel and it is possible to place $(c-1)/2$ disks. Also at most one disk with its center inside the node area can be placed but one disk may be placed and in this case, it is possible to place the disk with its center at *O*.^2^ Furthermore, the following holds.

- Only disks inside the channels and the node area can be added.
- If there is a disk whose center is properly inside the node area then at most $(c-1)/2$ disks can be placed in the left (right and bottom, respectively) channel. If exactly $(c-1)/2$ disks are placed, then one of the disks has its center at *X* (*Y* and *Z*, respectively).
- If there is a disk with its center at *U* (*V* and *W*, respectively) then at most $(c-1)/2$ disks (including the disk with its center at *U* (*V* and *W*, respectively)) can be placed in the left (right and bottom, respectively) channel, and if exactly $(c-1)/2$ disks are placed then they are completely inside the tile and it is impossible to place an additional disk having its center inside the tile except disks that may be placed in other channels.

### Proof

The proof immediately follows from Observations 3.6, 3.7, 3.11, and 3.12. $\square$

The construction of the node tile limits the choice of the constant *c* because for other tiles we need less space.

### Observation 3.14

The (straight, twisted, parity adjustment) channel, bend, and node tiles can be constructed for $c=47$.

### Proof

To construct the bottom channel in the node tile, we insert 7 gaps and a gap may be inserted between basic channels of size at least 2 (see Fig. 7a and Observation 3.7). Then taking into account the distance between the points *O* and *Q* in Fig. 12 and the number of gaps, we obtain that the bottom channel can be constructed for $c=47$. As for constructing the left and right channels in the node tile, we insert 3 gaps, we also have that they can be constructed for $c=47$. By similar arguments, we also can construct the (straight, twisted, parity adjustment) channel tile and the bend tile if $c=47$. $\square$

**Proof of** **Theorem** 1.1: **The final step.** Now we have all the ingredients to finish the hardness proof for Disk Appending problem.

Recall that we prove ${{\,\textrm{NP}\,}}$-hardness by reducing from Independent Set on planar cubic graphs [18, 28]. Let (*G*, *k*) be an instance of Independent Set where *G* is an *n*-vertex planar cubic graph. We would like to remind the reader of the initial steps. By Proposition 3.2, we can construct a rectilinear embedding of *G* with area $\mathcal {O}(n^2)$ in linear time. Further, we modify the embedding to ensure that the length of every segment of a broken line representing an edge in the embedding is at least three. As we already pointed out, this can be done by replacing every vertex or bend point (*x*, *y*) of the embedding with the point (3*x*, 3*y*) and the corresponding adjustment of the segments in the broken lines. After this modification, we still have an embedding with $\mathcal {O}(n^2)$ area. We assume that $R=[0,a]\times [0,b]$ for $a,b\in \mathbb {N}$ is the minimum area rectangle containing the embedding (note that $a,b>0$ because *G* is cubic and cannot be embedded on the line).

We define $a'=2ca$ and $b'=2cb$, where $c=47$, and set $R'=[0,a']\times [0,b']$ defining the rectangle in the output instance of Disk Appending problem. Then we put tiles into $R'$ as follows.

**Fig. 13:**
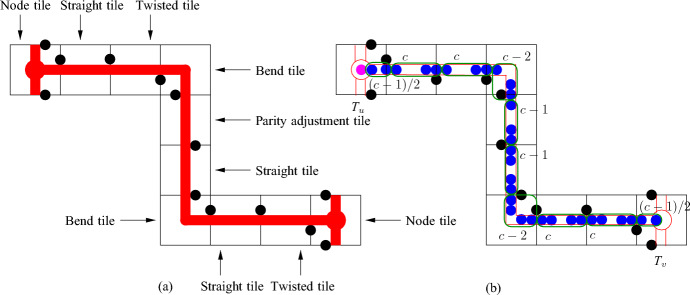
The construction of tiles for an edge and placement of tiles. The node areas and channels are shown in red and the poles are shown by black bullets. The placement of the disks in tiles associated with the edge is shown in blue and the disk that may be placed in the center of $T_u$ is shown in magenta

- For every $(x,y)\in R$ such that the point (*x*, *y*) is not a point of the embedding of *G*, put a copy of the filler tile whose bottom left corner in (2*cx*, 2*cy*).
- For every $(x,y)\in R$ such that (*x*, *y*) is a vertex of *G* in the embedding, put a copy of the node tile with the bottom left corner in (2*cx*, 2*cy*). We rotate the node tile in such a way that the directions of the left, right, and bottom channels coincide with the directions of line segments of the embedding with the endpoints in (*x*, *y*); note that because the distance between any two vertices in the embedding is at least three, the poles of distinct node tiles do not interfere with each other.
- For every $(x,y)\in R$ such that (*x*, *y*) is a bend node in the embedding of an edge, put a copy of the bend tile with the bottom left corner in (2*cx*, 2*cy*). We rotate the tile in such a way that the directions of the left and bottom channels coincide with the directions of line segments of the embedding with the endpoints in (*x*, *y*). Again we note that because the distance between a bend point and another bend point or a vertex in the embedding is at least three, there are no intersections between the poles of constructed tiles.
- For every edge $e\in E(G)$, let $P_e$ be the set of internal non-bending integer points of the embedding of *e*. We select an arbitrary point $(x,y)\in P_e$ and insert a copy *T* of the parity adjustment channel tile with the bottom left corner in (2*cx*, 2*cy*). We rotate *T* in such a way that the direction of its channel coincides with the direction of line segments of the embedding containing (*x*, *y*). Notice that because the length of every segment of a broken line representing an edge in the embedding is at least three, *T* may be adjacent to at most one already placed tile $T'$ whose pole intersects *T*. If such a pole of $T'$ has the same center as the corresponding pole of *T*, we unify these disks. Otherwise, if the poles have distinct centers, we mirror *T* to ensure that the poles have the same centers and unify them. For every other point $(x,y)\in P_e$, we insert a tile *T* which is a copy of either the straight or twisted channel tile. We rotate *T* to have the same direction of the channel as the direction of the segment of the line containing (*x*, *y*) in the embedding. Observe that *T* can have either one or two adjacent already placed tiles whose poles intersect *T*. If *T* is not adjacent to any such a tile, we select *T* to be a copy of the straight tile. If *T* is adjacent to one such tile $T'$ then we select *T* to be a copy of the straight channel tile. Then we either identify the interfering poles of *T* and $T'$ if they have the same centers or reflect *T* and identify the poles afterward. Suppose that *T* is adjacent to two tiles $T'$ and $T''$ with interfering poles. If the poles are on the same side with respect to the channel then we choose *T* to be a copy of the straight channel tile. Then we either identify the interfering poles of *T* and $T'$ if they have the same centers or reflect *T* and identify the poles afterward. Otherwise, we select *T* to be a copy of the twisted channel tile and reflect *T* if necessary to identify the poles. The construction of the tiles for $(x,y)\in P_e$ is shown in Fig. 13a.

We define $\mathcal {P}$ to be the set of all disks in the tiles (taking into account identifications of poles). By the construction, $\mathcal {P}$ is a packing of disks inside $R'$.

Clearly, we have *n* node tiles. Denote by $n_b$ the number of bend tiles, by $n_p=|E(G)|$ the number of parity adjustment channel tiles, and by $n_c$ the number of straight and twisted channel tiles. We set $k'=k+3n(c-1)/2+(c-2)n_b+(c-1)n_p+cn_c$.

We claim that *G* has an independent set of size of size at least *k* if and only if $(R',\mathcal {P},k')$ is a yes-instance of Disk Appending problem.

For every edge *e* of *G*, denote by $n_b^e$ the number of bend tiles and by $n_c^e$ the number of straight and twisted tiles in the set of tiles corresponding to the embedding of *e*.

For the forward direction, assume that *G* has an independent set *S* of size *k*. For every vertex $v\in S$, we consider the node tile $T_v$ corresponding to *v* and place a disk having its center at the center of the tile (point *O* in Fig. 12). Consider an edge $e=uv$ of *G*. Because *S* is an independent set $u\notin S$ or $v\notin S$. Assume without loss of generality that $v\notin S$ and it may happen that $u\in S$. Then we can insert $(c-1)/2$ disks in the channels of $T_v$ and $T_u$ corresponding to *e* by Lemma 3.13, $c-2$ disks per each bend tile by Lemma 3.10, $c-1$ disks in the unique parity adjustment channel tile by Lemma 3.8, and *c* disks per each straight or twisted channel tile by Lemma 3.5 as shown in Fig. 13b (we associate a pole disk shared by tiles with the first tile containing it along *e* if moving from *u* to *v*). Thus, we placed $2(c-1)/2+(c-2)n_b^e+(c-1)+cn_c^e$ disks. Summarizing over all edges and taking into account the disks corresponding to the vertices of *S*, we obtain that we placed $k'=k+3n(c-1)/2+(c-2)n_b+(c-1)n_p+cn_c$ disks.

For the opposite direction, assume that at least $k'$ disk can be placed in $R'$ to complement the packing $\mathcal {P}'$. By Lemmas 3.5 and 3.13, new disks can be only placed inside channels and node areas of the tiles. Let $\mathcal {S}$ be a packing of $k'$ disks in $R'$ disjoint with the disks of $\mathcal {P}$ such that the number of disks in $\mathcal {S}$ whose centers are properly inside of the node areas of node tiles is minimum.

Consider an edge $e=uv$ of *G*. By Lemmas 3.5 and 3.13, at most $2(c-1)/2+(c-2)n_b^e+(c-1)+cn_c^e$ disks can be placed in the channels of $T_v$ and $T_u$ corresponding to *e*, the bend tiles, the unique parity adjustment channel tile and all straight or twisted channel tiles (see Fig. 13b for an illustration). Moreover, for each $w\in V(G)$, at most one disk of $\mathcal {S}$ can have its center properly inside of the node area of $T_w$. Summarizing over all edges, we conclude that at least *k* disks of $\mathcal {S}$ have their centers in the node areas of node tiles, and for every $w\in V(G)$, at most one of these disks has its center inside of the node area of $T_w$.

Suppose that there are two disks in $\mathcal {S}$ such that their centers are inside of the node areas of $T_u$ and $T_v$. Then by Lemmas 3.5 and 3.13, we conclude that at most $2(c-1)/2+(c-2)n_b^e+(c-1)+cn_c^e-1$ disks are placed in the channels of $T_v$ and $T_u$ corresponding to *e* and other tiles associated with *e*. Then by Observation 3.11, we can relocate the disk with its center in the node area of $T_v$ and move it to the channel of $T_v$ associated with *e*. Then we still would be able to place $2(c-1)/2+(c-2)n_b^e+(c-1)+cn_c^e$ disks by the same arguments as in the proof for the forward direction. This means that the relocation does not decrease the number of added disks. However, this contradicts our assumption about the choice of $\mathcal {S}$, as we decrease the number of disks with centers that are properly inside the node areas of node tiles. Hence, for every $e=uv$, there is no disk with its center in the node area of $T_u$ or $T_v$.

Let $S\subseteq V(G)$ be the set of all vertices *w* such that the node tile $T_w$ has a disk of $\mathcal {S}$ with its center inside of the node area. We obtain that *S* is an independent set *G* of size at least *k*. This concludes the proof of our claim.

**Fig. 14:**
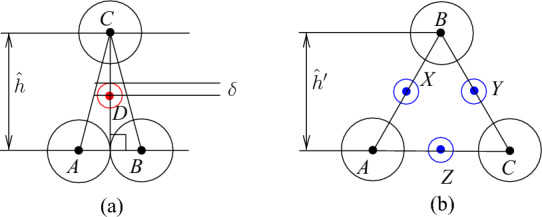
Rounding for the basic channels and node areas

This completes the description of the reduction and the correctness proof. However, we used disks with algebraic coordinates of their centers in the construction of the tiles. To fix this, we can observe that our construction is robust enough to allow rounding of coordinates. In particular, we can choose a sufficiently small constant $\delta >0$ and use rational parameters $\hat{h}$ and $\hat{h}'$ such that $2+\sqrt{3}=h<\hat{h}\le h+\delta$ and $2\sqrt{3}=h'<\hat{h}'\le h'+\delta$ in the construction of the basic channels (see Fig. 4) and the node areas (see Fig. 10a) instead of *h* and $h'$, respectively. Then for the crucial element of the construction of the basic channel, we can make the following observation. If the disks with their centers at *A*, *B*, and *C* are placed as shown in Fig. 14a then every disk with its center in the triangle *ABC* has its center at distance at most $\delta$ from a certain point *D*. For the node area, we can claim that if the disk with their centers at *A*, *B*, and *C* are placed as shown in Fig. 14b then it holds that if three other disks have centers in the triangle *ABC* then their centers are at distances at most $\delta$ from the centers of the sides of the triangle. These observations allow us to adjust the basic gadgets used in our reduction.

Similarly, we can round the parameters in the construction of the bend tiles (see Fig. 9a) and the node tiles (see Figs. 10a and  11). Furthermore, the construction of tiles (see Figs. 5, 9, and 12) allows us to accommodate the adjustments without changing the size of the tiles. For this, we may need to modify placing of the filler disks. We underline that all these adjustments are done for each type of tile and every tile contains at most $c^2$ disk. Therefore, each tile can be constructed in constant time.

To finish the proof of Theorem 1.1, note that the area of the rectilinear embedding of *G* constructed by the algorithm from Proposition 3.2 is $\mathcal {O}(n^2)$. Therefore, we construct $\mathcal {O}(n^2)$ tiles. Since the algorithm from Proposition 3.2 is polynomial, we conclude that the instance $(R',\mathcal {P},k')$ of Disk Appending problem constructed in polynomial time. This completes the proof of Theorem 1.1.

## An FPT Algorithm for Disk Repackingproblem

In this section, we give our algorithmic result on Disk Repacking problem. Recall that in an instance *I* of Disk Repacking problem, we are given a packing $\mathcal {P}$ of *n* unit disks inside a rectangle *R*, and two integers $h, k \ge 0$, and the task is to decide whether there exists a packing $\mathcal {P}^*$ of $n+k$ unit disks that is obtained from $\mathcal {P}$ by adding *k* new unit disks, and relocating at most *h* disks of $\mathcal {P}$ to new positions inside *R*. We show in the following (restated) theorem that Disk Repacking problem when parameterized by $k+h$.

### Theorem 4.1

The Disk Repacking problem is ${{\,\textrm{FPT}\,}}$ when parameterized by $k+h$. Specifically, it is solvable in time $(h+k)^{\mathcal {O}(h+k)}\cdot |I|^{\mathcal {O}(1)}$.

We first give an overview of the proof before giving the formal details.

**Proof of** **Theorem** 1.2: **Overview**. On a high-level, the idea behind the algorithm is as follows (in parentheses we also give relevant forward references to parts of the formal proof). We first perform a greedy procedure to ensure that all “free” areas to place disks can be intersected by a set $\mathcal H$ of at most *k* disks (Lemma 4.5). At this point, we want to use *color coding* to find a coloring function of $\mathcal P$, with the objective to color all disks in $\mathcal P$ that are repacked by a solution (if one exists) blue, and all disks in $\mathcal P$ that “closely surround” them by red. We need to ensure that, while relying on the initial greedy procedure, it would suffice to correctly color only $\mathcal {O}(h+k)$ disks. Indeed, this gives rise to the usage of a universal set, which is a “small” family of coloring functions ensured to contain, if there exists a solution, at least one coloring function that correctly colors all $\mathcal {O}(h+k)$ disks we care about (Lemma 4.9). Note that although color coding is a classical tool in the design of parameterized algorithms [2, 11]; the novelty of our algorithm is in coming up with the correct geometric definitions and objects that are suitable for the application of this tool.

Considering some coloring function (which is expected to be “compatible” with some hypothetical solution), we identify “slots” and, more generally, “containers” in its coloring pattern. In simple words, a slot is just a disk in *R* that does not intersect any red disk (from $\mathcal P$), and a container is a maximally connected region consisting of slots. We are able to prove that, if the coloring is compatible with some solution, then, for any container, either all or none of the disks in $\mathcal{P}$ that are contained in the container are repacked (Lemma 4.12). This gives rise to a reduction from the problem of finding a solution compatible with a given coloring to the Knapsack problem (more precisely, an extended version of it), where each container corresponds to an item whose weight is the number of disks in $\mathcal{P}$ that it contains (and thus, need to be repacked, as mentioned in the previous sentence), and whose value is the number of disks that can be packed within it. The goal of the Extended Knapsack problem is to find a subset of containers, whose total weight is some $W' \in \left\{ 0, 1, \ldots , h\right\}$, and whose total value is at least $W' + k$ (Lemma 4.23). Note that this corresponds to $W'$ disks in $\mathcal{P}$ contained in this subset being repacked (i.e., moved) within these containers, such that we can pack at least $W' + k$ disks within these containers – note that $W'$ out of these are the disks being moved within the containers, whereas *k* disks are being newly added.

To execute the reduction described above, we need to be able to compute the value of each container. For this purpose, we first prove that a container can be “described” by only $\mathcal {O}(h+k)$ many disks from $\mathcal{P}\cup \mathcal{H}$; more precisely, we show that each container is the union of disks contained in *R* that intersect at least one out of $\mathcal {O}(h+k)$ disks in $\mathcal{P}\cup \mathcal{H}$, from which we subtract the union of some other $\mathcal {O}(h+k)$ disks from $\mathcal{P}$ (Lemma 4.16). Having this at hand, to compute the value of a container, we first “guess”, for each disk packed by a (hypothetical) optimal packing of disks in the container, a disk from $\mathcal{P}\cup \mathcal{H}$ contained in the container (making use of its description) with whom it intersects. After that, we seek the corresponding optimal packing by solving systems of polynomial inequalities of degree 2, with $\mathcal {O}(h+k)$ variables, and $\mathcal {O}((h+k)^2)$ equations (Lemma 4.17).

**Proof of** **Theorem** 1.2:**Free Areas.** To execute the plan above, we start with the task of handling the “free” areas. For this, we have the following definition and an immediate observation.

### Definition 4.2

[**Holes and Hole Cover**] Let $(\mathcal{P}, R,h,k)$ be an instance of Disk Repacking problem. The *set of holes*, denoted by $\textsf{Holes}$, is the set of all unit disks contained in *R* that are disjoint from all unit disks in $\mathcal P$. A set $\mathcal{H}$ of unit disks contained in *R* such that the set of holes of $(\mathcal{P}\cup \mathcal{H}, R,h,k)$ is empty is called a *hole cover*.

**Fig. 15:**
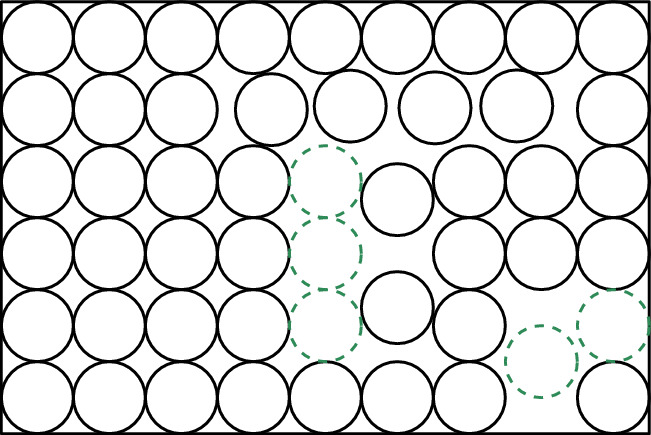
An instance $(\mathcal {P}, R, h = 2, k = 7)$ of Disk Repacking problem. The disks in $\mathcal {P}$ are colored black. The disks in some hole cover $\mathcal {H}$ are colored green (using dashed lines)

**Fig. 16:**
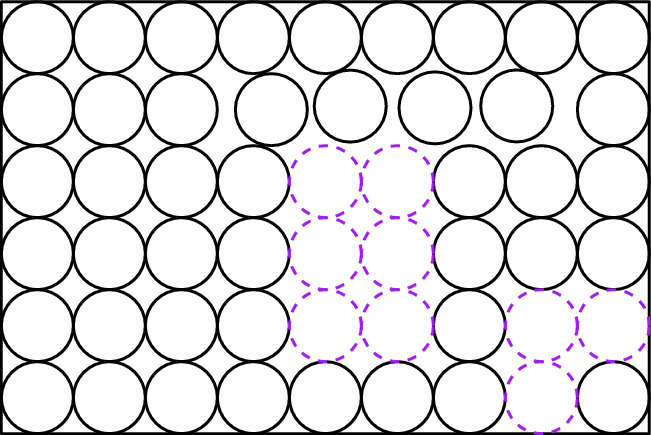
A solution $\mathcal {P}^*$ for the instance on the left. The disks in $\mathcal {P}^* \setminus \mathcal {P}$ are drawn in purple (using dashed lines).The set of $(\mathcal {H}, \mathcal {P}^*)$-critical disks is the set of green disks from the figure on the left and the purple disks from the figure on the right

**Fig. 17:**
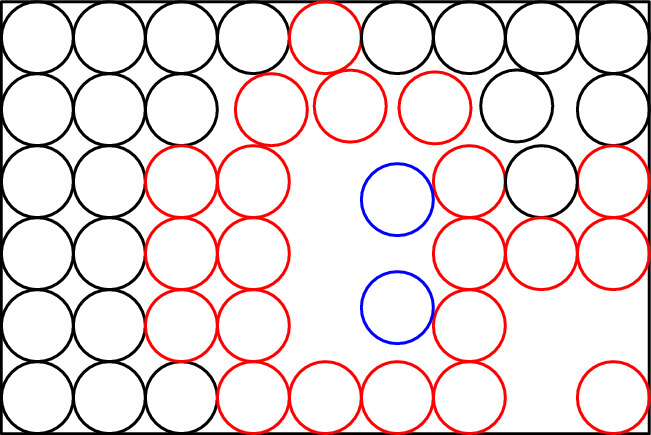
With respect to the instance and solution described in Figs. 15 and 16, the disks $(\mathcal {H}, \mathcal {P}^*)$-forced to be blue are colored blue, and the disks $(\mathcal {H},\mathcal {P}^*)$-forced to be red are colored red. Note that each of the disks colored black can be colored either blue or red by an $(\mathcal {H},\mathcal {P}^*)$-compatible coloring

**Fig. 18:**
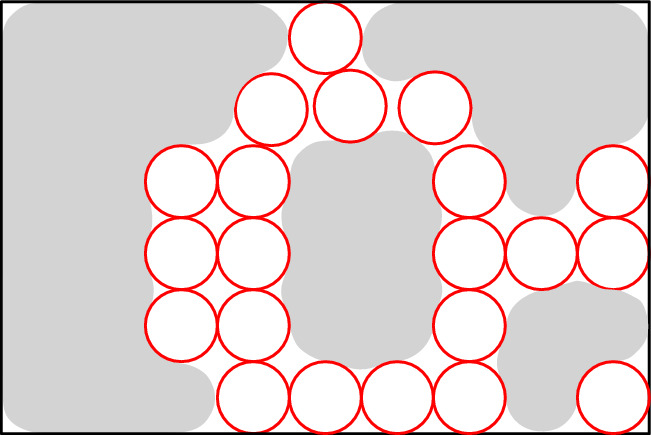
Consider an $(\mathcal {H}, \mathcal {P}^*)$-compatible coloring that colors blue all of the disks colored black in Fig. 17. Then, we have four *c*-Containers, which roughly correspond to the areas colored by grey

### Observation 4.3

Let $(\mathcal{P}, R,h,k)$ be an instance of Disk Repacking problem. Let $\mathcal{H}$ be a hole cover. Then, every disk contained in *R* intersects at least one disk in $\mathcal{P}\cup \mathcal{H}$.

Next, we present a definition and a lemma that will allow us to assume that there is always a hole cover of small size at hand.

### Definition 4.4

[**Dense instance**] Let $(\mathcal{P}, R,h,k)$ be an instance of Disk Repacking problem. We say that the instance is *dense* if it has a hole cover of size smaller than *k*.

### Lemma 4.5

There exists a polynomial-time algorithm that, given an instance $(\mathcal{P}, R,h,k)$ of Disk Repacking problem, either correctly determines that $(\mathcal{P}, R,h,k)$ is a yes-instance or correctly determines that $(\mathcal{P}, R,h,k)$ is dense and returns a hole cover of size smaller than *k*.

### Proof

We perform a simple greedy procedure. Initially, $\mathcal{H}=\emptyset$. Then, as long as there exists a disk *D* contained in *R* that is disjoint from all disks in $\mathcal{P}\cup \mathcal{H}$, we add such a disk *D* to $\mathcal H$. The test for the existence of such a *D* can be performed by using a system of polynomial equations of degree 2 with two variables denoting the *x*- and *y*-coordinates of the center of *D*. For each disk in $\mathcal{P}\cup \mathcal{H}$, we have an equation enforcing that the distance between its center and the center of *D* is at least 2, and additionally we have two linear equations to enforce that *D* is contained in *R*. By Proposition 2.1, testing whether this system has a solution (which corresponds to the sought disk *D*) can be done is polynomial time. Once the process terminates, the algorithm checks whether $|\mathcal{H}|\ge k$. If the answer is positive, then adding $\mathcal{H}$ (or, more precisely, any subset of size *k* of it) to $\mathcal P$ is a solution, and so the algorithm answers yes, and otherwise the instance is dense and the algorithm returns $\mathcal H$ (which witnesses that). $\square$

In the two following definitions, we identify the coloring functions that will be useful.

### Definition 4.6

[ $(\mathcal{H},\mathcal{P}^*)$-**Critical Disks**] Let $(\mathcal{P}, R,h,k)$ be a yes-instance of Disk Repacking problem. Let $\mathcal{H}$ be a hole cover. Let $\mathcal{P}^*$ be a solution to $(\mathcal{P}, R,h,k)$. The set of $(\mathcal{H},\mathcal{P}^*)$-*critical disks*, denoted by $\textsf{Crit}_{\mathcal{H},\mathcal{P}^*}$, is $(\mathcal{P}^*{\setminus } \mathcal{P})\cup \mathcal{H}$.

### Definition 4.7

[$(\mathcal{H},\mathcal{P}^*)$-**Compatible Colorings**] Let $(\mathcal{P}, R,h,k)$ be a yes-instance of Disk Repacking problem. Let $\mathcal{H}$ be a hole cover. Let $\mathcal{P}^*$ be a solution to $(\mathcal{P}, R,h,k)$. Let $c: \mathcal{P}\rightarrow \{\textsf{blue},\textsf{red}\}$. We say that *c* is $(\mathcal{H},\mathcal{P}^*)$-*compatible* if:

1. For every $D\in \mathcal{P}\setminus \mathcal{P}^*$, we have that $c(D)=\textsf{blue}$. We say that the disks in $\mathcal{P}\setminus \mathcal{P}^*$ are $(\mathcal{H},\mathcal{P}^*)$-*forced to be blue*.
2. For every $D\in \mathcal{P}\cap \mathcal{P}^*$ whose center is at distance at most 4 from the center of some disk in $\textsf{Crit}_{\mathcal{H},\mathcal{P}^*}$, we have that $c(D)=\textsf{red}$. We say that the disks in $\mathcal{P}\cap \mathcal{P}^*$ whose center is at distance at most 4 from the center of some disk in $\textsf{Crit}_{\mathcal{H},\mathcal{P}^*}$ are $(\mathcal{H},\mathcal{P}^*)$-*forced to be red*.

We proceed to show that the number of disks in $\mathcal P$ that should be colored “correctly” is only $\mathcal {O}(h+k)$. This is done using the following easy observation, in the following lemma.

### Observation 4.8

The number of pairwise disjoint disks inside a circle of radius *r* is at most $r^2$.

### Lemma 4.9

Let $(\mathcal{P}, R,h,k)$ be a dense yes-instance of Disk Repacking problem. Let $\mathcal{H}$ be a hole cover of size smaller than *k*. Let $\mathcal{P}^*$ be a solution to $(\mathcal{P}, R,h,k)$. Then, the number of disks $(\mathcal{H},\mathcal{P}^*)$-forced to be either blue or red is altogether bounded by $\mathcal {O}(h+k)$.

### Proof

Because $\mathcal{P}^*$ is a solution and $|\mathcal{H}|<k$, we have that $|\mathcal{P}\setminus \mathcal{P}^*|\le h$. So, at most *h* disks are $(\mathcal{H},\mathcal{P}^*)$-forced to be blue. Further, $|\textsf{Crit}_{\mathcal{H},\mathcal{P}^*}|=|(\mathcal{P}^*{\setminus } \mathcal{P})\cup \mathcal{H}|<h+2k$. Observe that every disk in $\mathcal{P}\cap \mathcal{P}^*$ whose center is at distance at most 4 from the center of some disk in $\textsf{Crit}_{\mathcal{H},\mathcal{P}^*}$ is contained inside a circle of radius 5 whose center is the center of some disk in $\textsf{Crit}_{\mathcal{H},\mathcal{P}^*}$. So, due to Observation 4.8 and since the disks in $\mathcal{P}\cap \mathcal{P}^*$ are pairwise disjoint, there exist at most $\pi \cdot 5^2\cdot (h+2k)=\mathcal {O}(h+k)$ disks in $\mathcal{P}\cap \mathcal{P}^*$ whose center is at distance at most 4 from the center of some disk in $\textsf{Crit}_{\mathcal{H},\mathcal{P}^*}$. In particular, this means that at most $\mathcal {O}(h+k)$ disks are $(\mathcal{H},\mathcal{P}^*)$-forced to be red.

This completes the proof. $\square$

**Proof of** **Theorem** 1.2:**Values of Containers.** Next, we present the definition of slots and containers, in which we will aim to (re)pack disks. The definition is followed by an observation and a lemma, which, in particular, state that if we try to repack at least one disk in a container, we can just repack all disks in that container.

### Definition 4.10

[ *c* **-Slots and** *c* **-Containers**] Let $(\mathcal{P}, R,h,k)$ be an instance of Disk Repacking problem. Let $c: \mathcal{P}\rightarrow \{\textsf{blue},\textsf{red}\}$. The set of *c-slots*, denoted by $\textsf{Slots}_c$, is the set of disks contained in *R* that are disjoint from all disks in $\mathcal P$ that are colored red by *c*. The set of *c*-*containers*, denoted by $\textsf{Containers}_c$, is the set of maximally connected regions in the union of all disks in $\textsf{Slots}_c$.

### Observation 4.11

Let $(\mathcal{P}, R,h,k)$ be an instance of Disk Repacking problem. Let $c: \mathcal{P}\rightarrow \{\textsf{blue},\textsf{red}\}$. Then, the regions in $\textsf{Containers}_c$ are pairwise disjoint.

### Lemma 4.12

Let $(\mathcal{P}, R,h,k)$ be a yes-instance of Disk Repacking problem. Let $\mathcal{H}$ be a hole cover. Let $\mathcal{P}^*$ be a solution to $(\mathcal{P}, R,h,k)$. Let $c: \mathcal{P}\rightarrow \{\textsf{blue},\textsf{red}\}$ be $(\mathcal{H},\mathcal{P}^*)$-compatible. Then, for every region $X\in \textsf{Containers}_c$, either all disks in $\mathcal{P}$ contained in *X* belong to $\mathcal{P}\setminus \mathcal{P}^*$ or none of the disks in $\mathcal{P}\cup \mathcal{P}^*$ contained in *X* belongs to $(\mathcal{P}\setminus \mathcal{P}^*)\cup (\mathcal{P}^*\setminus \mathcal{P})$.^3^

### Proof

Aiming for a contradiction, suppose that there exists a disk *D* contained in *X* that belongs to $(\mathcal{P}{\setminus }\mathcal{P}^*)\cup (\mathcal{P}^*{\setminus }\mathcal{P})$ and a disk $D'$ contained in *X* that belongs to $\mathcal{P}\cap \mathcal{P}^*$. Let $\gamma$ be a curve connecting the centers of these disks that lies entirely inside *X*. By the definition of a *c*-container and due to Observation 4.3, every point of this curve contained in a disk that belongs to *X* and intersects a disk in $\mathcal P$ colored blue by *c* or a disk in $\mathcal H$. So, there must exist a point on $\gamma$ that is the center of a disk $D^*$ that intersects both a disk *A* contained in *X* that belongs to $(\mathcal{P}{\setminus }\mathcal{P}^*)\cup \mathcal{H}$ and a disk $A'$ contained in *X* that belongs to $\mathcal{P}\cap \mathcal{P}^*$. From the definition of a *c*-container, $A'$ is colored blue by *c*. Moreover, note that the center of $A'$ is at distance at most 4 from the center of *A*, since each of the centers of *A* and $A'$ is at distance at most 2 from the center of $D^*$. However, since *c* is $(\mathcal{H},\mathcal{P}^*)$-compatible, $A'$ is $(\mathcal{H},\mathcal{P}^*)$-forced to be red and hence it is colored red by *c*. Since *c* cannot color a disk both blue and red, we have reached a contradiction. This completes the proof. $\square$

We proceed to define the weight and value of a *c*-container, which will be required for the reduction of our problem to Knapsack.

### Definition 4.13

[**Weight, Validity and Value of Containers**] Let $(\mathcal{P}, R,h,k)$ be an instance of Disk Repacking problem. Let $c: \mathcal{P}\rightarrow \{\textsf{blue},\textsf{red}\}$. Let $X\in \textsf{Containers}_c$. The *weight* of *X* is the number of disks in $\mathcal P$ that it contains.

We say that *X* is *valid* if its weight is at most *h*. The *value* of *X* is the maximum number of disks that can be packed inside *X*.

The following is a corollary of Lemma 4.12.

### Corollary 4.14

Let $(\mathcal{P}, R,h,k)$ be a yes-instance of Disk Repacking problem. Let $\mathcal{P}^*$ be a solution to $(\mathcal{P}, R,h,k)$. Let $c: \mathcal{P}\rightarrow \{\textsf{blue},\textsf{red}\}$ be $(\mathcal {H},\mathcal {P}^*)$-compatible. Then, every disk in $(\mathcal{P}{\setminus }\mathcal{P}^*)\cup (\mathcal{P}^*{\setminus }\mathcal{P})$ is a *c*-slot, and it is contained in a valid *c*-container.

Now, we define how to “easily” describe a container, and then prove that this description be encoded compactly.

### Definition 4.15

[**Descriptions of Containers**] Let $(\mathcal{P}, R,h,k)$ be an instance of Disk Repacking problem. Let $\mathcal{H}$ be a hole cover. Let $c: \mathcal{P}\rightarrow \{\textsf{blue},\textsf{red}\}$. An $\mathcal H$-*description* (or, for short, *description*) of a region $X\in \textsf{Containers}_c$ is a pair $(\mathcal{D}_1,\mathcal{D}_2)$ of a subset $\mathcal{D}_1\subseteq \mathcal{P}\cup \mathcal{H}$ and a minimal subset $\mathcal{D}_2\subseteq \mathcal{P}$ such that *X* equals the set of all points in *R* at distance less than 2 from at least one disk in $\mathcal{D}_1$ and at least 2 from all disks in $\mathcal{D}_2$.

### Lemma 4.16

Let $(\mathcal{P}, R,h,k)$ be an instance of Disk Repacking problem. Let $\mathcal{H}$ be a hole cover. Let $c: \mathcal{P}\rightarrow \{\textsf{blue},\textsf{red}\}$. Let $X\in \textsf{Containers}_c$. Then, *X* has at least one description $(\mathcal{D}_1,\mathcal{D}_2)$. Moreover, every description $(\mathcal{D}_1,\mathcal{D}_2)$ of *X* satisfies $|\mathcal{D}_1|+|\mathcal{D}_2|=\mathcal {O}(h'+k')$ where $h'$ is the weight of *X*, and $k'$ is the number of disks in $\mathcal H$ contained in *X*.

### Proof

By Observation 4.3, every *c*-slot intersects at least one disk in $\{D\in \mathcal{P}: c(D)=\textsf{blue}\}\cup \mathcal{H}$ and is disjoint from all disks in $\{D\in \mathcal{P}: c(D)=\textsf{red}\}$. Further, every point in every disk in $\{D\in \mathcal{P}: c(D)=\textsf{blue}\}\cup \mathcal{H}$ is contained in a *c*-slot. So, it is immediate that *X* has a description $(\mathcal{D}_1,\mathcal{D}_2)$, and that $|\mathcal{D}_1|=\mathcal {O}(h'+k')$. Due to Observation 4.8 and since the disks in $\mathcal{P}\cup \mathcal{H}$ are pairwise disjoint, any circle of radius 5 whose center is a center of some disk in $\{D\in \mathcal{P}: c(D)=\textsf{blue}\}\cup \mathcal{H}$ can contain inside at most $5^2$ disks from $\{D\in \mathcal{P}: c(D)=\textsf{red}\}$. Due to the minimality of $\mathcal{D}_2$ (which is a subset of $\{D\in \mathcal{P}: c(D)=\textsf{red}\}$), every disk in it must be contained inside a circle of radius 5 whose center is a center of some disk in $\{D\in \mathcal{P}: c(D)=\textsf{blue}\}\cup \mathcal{H}$. Hence, $|\mathcal{D}_2|\le |\mathcal{D}_1| \cdot 5^2=\mathcal {O}(h'+k')$. $\square$

Next, we use a description in order to efficiently compute the value of a *c*-container.

### Lemma 4.17

There is an $(h+k)^{\mathcal {O}(h+k)}\cdot |I|^{\mathcal {O}(1)}$-time algorithm that, given a dense instance $I=(\mathcal{P}, R,h,k)$ of Disk Repacking problem, a hole cover $\mathcal{H}$ of size smaller than *k*, $c: \mathcal{P}\rightarrow \{\textsf{blue},\textsf{red}\}$ and a valid region *X* with a description $(\mathcal{D}_1,\mathcal{D}_2)$, computes the value of *X*.

### Proof

Given $I=(\mathcal{P}, R,h,k), \mathcal{H}, c, X$ and $(\mathcal{D}_1,\mathcal{D}_2)$, the algorithm works as follows. For $\ell =h+k,h+k-1,\ldots ,1$, and for every vector $(D_1,D_2,\ldots ,D_\ell )\in \mathcal{D}_1\times \mathcal{D}_1\times \cdots \times \mathcal{D}_1$, it tests whether there exist $\ell$ disks $S_1,S_2,\ldots ,S_\ell$ such that, for every $i\in \{1,2,\ldots ,\ell \}$, $S_i$ intersects $D_i$, is contained in *R* and is disjoint from all disks in $\mathcal{D}_2$. The test is done by constructing a system of polynomial equations of degree 2 with $2\ell$ variables and $\ell \cdot (|\mathcal{D}_2|+2)$ equations as follows. For every $i\in \{1,2,\ldots ,\ell \}$, we have two variables, denoting the *x*- and *y*-coordinates of the center of $S_i$, one equation enforcing that the distance between the center of $S_i$ and the center of $D_i$ is smaller than 2, $|\mathcal{D}_2|$ equations enforcing that the distance between the center of $S_i$ and the center of each of the disks in $\mathcal{D}_2$ is at least 2, and two linear equations enforcing that $S_i$ is contained inside *R*. If the answer is positive, then the algorithm returns that the value of *X* is $\ell$ and terminates; else, it proceeds to the next iteration. Observe that, when $\ell =1$, the algorithm necessarily terminates (since *X* contains at least one *c*-slot).

The correctness of the algorithm is immediate from the definition of a description and the exhaustive search that it performs. As for the running time, first observe that, by Lemma 4.16 and since *X* is valid and $|\mathcal{H}|<k$, $|\mathcal{D}_1|+|\mathcal{D}_2|\le \mathcal {O}(h+k)$. So, for a given $\ell$, we have $|\mathcal{D}_1|^{\mathcal {O}(\ell )}=(h+k)^{\mathcal {O}(h+k)}$ choices of vectors. Now, consider the iteration corresponding to some $\ell$ and some vector. Then, we solve a system of polynomial equations of degree 2 with $\mathcal {O}(h+k)$ variables and $\mathcal {O}((h+k)^2)$ equations. By Proposition 2.1, this can be done in time $(h+k)^{\mathcal {O}(h+k)}\cdot |I|^{\mathcal {O}(1)}$. Thus, the algorithm indeed runs in time $(h+k)^{\mathcal {O}(h+k)}\cdot |I|^{\mathcal {O}(1)}$. $\square$

The following definition captures the set of all descriptions.

### Definition 4.18

[**Blueprint**] Let $(\mathcal{P}, R,h,k)$ be an instance of Disk Repacking problem. Let $\mathcal{H}$ be a hole cover. Let $c: \mathcal{P}\rightarrow \{\textsf{blue},\textsf{red}\}$. An $(\mathcal{H},c)$-*blueprint* is

a collection of pairs of sets $\textsf{Blueprint}\subseteq 2^{\mathcal{P}\cup \mathcal{H}}\times 2^\mathcal{{P}}$, where the first elements of the pair are pairwise-disjoint subsets of $\mathcal {P}\cup \mathcal {H}$, such that each region in $\textsf{Containers}_c$ has exactly one description in $\textsf{Blueprint}$, and every pair in $\textsf{Blueprint}$ is a description of a region in $\textsf{Containers}_c$.

Next, we show how to compute blueprints.

### Lemma 4.19

There exists a polynomial-time algorithm that, given an instance $(\mathcal{P}, R,h,k)$ of Disk Repacking problem,

a hole cover $\mathcal{H}$, and $c: \mathcal{P}\rightarrow \{\textsf{blue},\textsf{red}\}$, outputs an $(\mathcal{H},c)$-blueprint.

### Proof

We will perform a simple greedy procedure to identify, for each disk in $\{D\in \mathcal{P}: c(D)=\textsf{blue}\}\cup \mathcal{H}$, the description of the region that contains it. Observe that every *c*-container contains at least one disk in $\{D\in \mathcal{P}: c(D)=\textsf{blue}\}\cup \mathcal{H}$ (due to Observation 4.3 and the definition of a *c*-container). So, for every disk $D\in \{D\in \mathcal{P}: c(D)=\textsf{blue}\}\cup \mathcal{H}$ such that we have not already taken a description of a region that contains it,,^4^ we will take exactly one description $(\mathcal{D}_1,\mathcal{D}_2)$ among the descriptions we identified such that *D* is contained in $\mathcal{D}_1$. Thus, we will obtain an $(\mathcal{H},c)$-blueprint.

To describe the greedy procedure, consider some $D\in \{D\in \mathcal{P}: c(D)=\textsf{blue}\}\cup \mathcal{H}$. Let us first show how to attain $\mathcal{D}_1$. For this purpose, we initialize $\mathcal{D}_1=\{D\}$. Then, for every pair of disks $A\in \mathcal{D}_1$ and $B\in (\{D\in \mathcal{P}: c(D)=\textsf{blue}\}\cup \mathcal{H}){\setminus }\mathcal{D}_1$, we test whether there exists a pair of disks *C* and $C'$ that are contained in *R*, intersect each other, are disjoint from all disks in $\{D\in \mathcal{P}: c(D)=\textsf{red}\}$, and such that *C* intersects *A* and $C'$ intersects *B*. The test for the existence of such a *C* is performed by using a system of polynomial equations of degree 2 with four variables denoting the *x*- and *y*-coordinates of the centers of *C* and $C'$. For each disk in $\{D\in \mathcal{P}: c(D)=\textsf{red}\}$, we have two equations enforcing that the distances between its center and the centers of *C* and $C'$ are each at least 2. Additionally, we have three equations to enforce that the distance between the centers of *C* and $C'$ is smaller than 2, the distance between the centers of *C* and *A* is smaller than 2, and the distance between the centers of $C'$ and *B* is smaller than 2, as well as four linear equations to enforce that *C* and $C'$ are contained in *R*. By Proposition 2.1, testing whether this system has a solution (which corresponds to the sought disks *C* and $C'$) can be done is polynomial time. If the answer is positive, then we add *B* to $\mathcal{D}_1$. In case at least one pair (*A*, *B*) resulted in the addition of *B* to $\mathcal{D}_1$, then we repeat the entire loop, iterating again over all pairs (*A*, *B*) (where the domain from which they are taken is updated as a new disk was added to $\mathcal{D}_1$). Notice that we can perform at most $|\mathcal{P}|$ repetitions, and that each repetition results in at most $|\mathcal{P}\cup \mathcal{H}|^2$ many iterations, each taking polynomial time. Hence, the procedure, so far, runs in polynomial time.

Now, let us show how to attain $\mathcal{D}_2$. For this purpose, we initialize $\mathcal{D}_2=\{D\in \mathcal{P}: c(D)=\textsf{red}\}$. Now, for every $A\in \{D\in \mathcal{P}: c(D)=\textsf{red}\}$, we test whether there exists a disk *C* that is contained in *R* and intersects both *A* and at least one disk in $\mathcal{D}_1$, and is disjoint from all disks in $\mathcal{D}_2\setminus \{A\}$. The test can be performed by iterating over every disk $B\in \mathcal{D}_1$, and using a system of polynomial equations of degree 2 with two variables denoting the *x*- and *y*-coordinates of the center of *C*. For each disk in $\mathcal{D}_2\setminus \{A\}$, we have an equation enforcing that the distance between its center and the center of *C* is at least 2, and additionally we have two equations to enforce that the distance between the center of *C* and each of the centers of *A* and *B* is smaller than 2, as well as two linear equations to enforce that *C* is contained in *R*. By Proposition 2.1, testing whether this system has a solution (which corresponds to the sought disk *C*) can be done is polynomial time. If the answer is positive, then we remove *A* from $\mathcal{D}_2$. Notice that this phase of the procedure also runs in polynomial time. Moreover, the correctness of the entire procedure directly follows from the definitions of a *c*-container and a description. $\square$

We proceed to define the (extended version of the) Knapsack problem and the instances of this problem that our reduction produces.

### Definition 4.20

[**(Extended) Knapsack**] In the (Extended) Knapsack problem, we are given a collection of *n* items *U*, where each item $u\in U$ has a weight $w(u)\in \mathbb {N}_0$ and a value $v(u)\in \mathbb {N}_0$, and an integer $W\in \mathbb {N}_0$. The objective is to find, for every $W'\in \{0,1,\ldots ,W\}$, the maximum $V_{W'}\in \mathbb {N}_0$ for which there exists a subset of items $S\subseteq \{1,2,\ldots ,n\}$ such that $\sum _{i\in S}w(u)\le W'$ and $\sum _{i\in S}v(u)\ge V_{W'}$. Such an instance of Knapsack is denoted by the tuple (*U*, *w*, *v*, *W*).

### Definition 4.21

[$(\mathcal{H},c)$-Knapsack**instance**] Let $(\mathcal{P}, R,h,k)$ be an instance of Disk Repacking problem. Let $\mathcal{H}$ be a hole cover. Let $c: \mathcal{P}\rightarrow \{\textsf{blue},\textsf{red}\}$. The $(\mathcal{H},c)$-Knapsack *instance* is the instance (*U*, *w*, *v*, *W*) of Knapsack defined as follows: *U* is the set of all valid regions in $\textsf{Containers}_c$; for each $X\in U$, *w*(*X*) and *v*(*X*) are the weight and value of *X* (see Definition 4.13); $W=h$.

### Proposition 4.22

[9] The (Extended) Knapsack problem is solvable in time $\mathcal {O}(|U|\cdot W)$.

We now to prove the correspondence between our problem when we restrict the solution set to solutions compatible with a given coloring and the Knapsack problem.

### Lemma 4.23

Let $(\mathcal{P}, R,h,k)$ be an instance of Disk Repacking problem. Let $\mathcal{H}$ be a hole cover. Let $c: \mathcal{P}\rightarrow \{\textsf{blue},\textsf{red}\}$. Then, there exists a solution $\mathcal{P}^*$ to $(\mathcal{P}, R,h,k)$ such that *c* is compatible with $\mathcal{P}^*$ if and only if for the $(\mathcal{H},c)$-Knapsack instance (*U*, *w*, *v*, *W*), there exists $W'\in \{0,1,\ldots ,W\}$ such that $V_{W'}\ge W'+k$.

### Proof

In one direction, suppose that there exists a solution $\mathcal{P}^*$ to $(\mathcal{P}, R,h,k)$ such that *c* is compatible with $\mathcal{P}^*$. Let $X_1,X_2,\ldots ,X_\ell$ be the *c*-containers that contain at least one disk from $(\mathcal{P}{\setminus }\mathcal{P}^*)\cup (\mathcal{P}^*{\setminus }\mathcal{P})$. Observation 4.11 implies that these *c*-containers are pairwise disjoint. By Lemma 4.12, and since *c* is compatible with $\mathcal{P}^*$, all disks in $\mathcal{P}$ contained in $X_1\cup X_2\cup \cdots \cup X_\ell$ belong to $\mathcal{P}{\setminus }\mathcal{P}^*$. Finally, by Corollary 4.14 and since *c* is compatible with $\mathcal{P}^*$, all disks in $(\mathcal{P}{\setminus }\mathcal{P}^*)\cup (\mathcal{P}^*{\setminus }\mathcal{P})$ are contained in $X_1\cup X_2\cup \cdots \cup X_\ell$, and all of these *c*-containers are valid. So, because $\mathcal{P}^*$ can repack *h* disks from $\mathcal P$, the total weight of these *c*-containers must be some $W'\in \{0,1,\ldots ,h\}=\{0,1,\ldots ,W\}$, and since $\mathcal{P}^*$ also packs *k* additional disks, the total value of these *c*-containers must be at least $W'+k$ (to accommodate all of the repacked and *k* newly packed disks). Thus, $V_{W'}\ge W'+k$.

In the other direction, suppose that there exists $W'\in \{0,1,\ldots ,W\}$ such that $V_{W'}\ge W'+k$. This means that there exist *c*-containers $X_1,X_2,\ldots ,X_\ell$ whose total weight is $W'\in \{0,1,\ldots ,h\}$ and whose total value is at least $W'+k$. However, because these *c*-containers are pairwise disjoint (by Observation 4.11), this means that we can construct a solution $\mathcal{P}^*$ such that *c* is compatible with $\mathcal{P}^*$ by repacking all the disks in $\mathcal P$ that are contained in $X_1,X_2,\ldots ,X_\ell$ (there are at most *h* such disks) and, additionally, inserting *k* new disks, within $X_1,X_2,\ldots ,X_\ell$. This completes the proof. $\square$

The following is a corollary of Lemmas 4.17 and 4.19.

### Corollary 4.24

There exists an $(h+k)^{\mathcal {O}(h+k)}\cdot |I|^{\mathcal {O}(1)}$-time algorithm that, given a dense instance $I=(\mathcal{P}, R,h,k)$ of Disk Repacking problem, a hole cover $\mathcal{H}$ of size smaller than *k* and $c: \mathcal{P}\rightarrow \{\textsf{blue},\textsf{red}\}$, computes the $(\mathcal{H},c)$-Knapsack instance.

To compute coloring functions, we will use the following definition and proposition.

### Definition 4.25

[(*U*, *k*)-**Universal Set**] For a universe *U* and $k\in \mathbb {N}$, a (*U*, *k*)-*universal set* is a collection $\mathcal C$ of functions $f: U\rightarrow \{\textsf{blue},\textsf{red}\}$ such that for every pair of disjoint sets $B,R\subseteq U$ whose union has size at most *k*, there exists $c\in \mathcal{C}$ that colors all integers in *B* blue and all integers in *R* red.

### Proposition 4.26

[29] There exists an algorithm that, given a universe *U* of size *n* and $k\in \mathbb {N}$, constructs a (*U*, *k*)-universal set of size $2^{k+\mathcal {O}(\log ^2 k)}\log n$ in time $2^{k+\mathcal {O}(\log ^2 k)}n\log n$.

Based on the definition of a universal set, we define the collection of Knapsack instances relevant to our reduction.

### Definition 4.27

[$(\mathcal{H},\mathcal{C})$-Knapsack **Collection**] Let $(\mathcal{P}, R,h,k)$ be an instance of Disk Repacking problem. Let $\mathcal{H}$ be a hole cover. Let $\mathcal{C}$ be a $(\mathcal{P},q(h+k))$-universal set, where *q* is the constant hidden in the $\mathcal {O}$-notation in Lemma 4.9. Then, the $(\mathcal{H},\mathcal{C})$-Knapsack*collection* is the collection of Knapsack instances that includes, for every $c\in \mathcal{C}$, the $(\mathcal{H},c)$-Knapsack instance.

The following is a corollary of Corollary 4.24.

### Corollary 4.28

There exists an $(h+k)^{\mathcal {O}(h+k)}\cdot |I|^{\mathcal {O}(1)}$-time algorithm that, given a dense instance $I=(\mathcal{P}, R,h,k)$ of Disk Repacking problem, a hole cover $\mathcal{H}$ of size smaller than *k* and a $(\mathcal{P},q(h+k))$-universal set $\mathcal C$, computes the $(\mathcal{H},\mathcal{C})$-Knapsack collection.

Next, we prove the correspondence between our problem and the collection of Knapsack instances we have just defined.

### Lemma 4.29

Let $(\mathcal{P}, R,h,k)$ be an instance of Disk Repacking problem. Let $\mathcal{H}$ be a hole cover. Let $\mathcal{C}$ be a $(\mathcal{P},q(h+k))$-universal set. Then, $(\mathcal{P}, R,h,k)$ is a yes-instance of Disk Repacking problem and only if the $(\mathcal{H},\mathcal{C})$-Knapsack collection contains an instance (*U*, *w*, *v*, *W*, *V*) for which there exists $W'\in \{0,1,\ldots ,W\}$ such that $V_{W'}\ge W'+k$.

### Proof

In one direction, suppose that $(\mathcal{P}, R,h,k)$ is a yes-instance. By the definition of a $(\mathcal{P},q(h+k))$-universal set and due to Lemma 4.9, there exists $c\in \mathcal{C}$ that is compatible with $\mathcal{P}^*$. So, the $(\mathcal{H},c)$-Knapsack instance is contained in the $(\mathcal{H},\mathcal{C})$-Knapsack collection (*U*, *w*, *v*, *W*, *V*), and by Lemma 4.23, for this instance there exists $W'\in \{0,1,\ldots ,W\}$ such that $V_{W'}\ge W'+k$.

In the other direction, suppose that the $(\mathcal{H},\mathcal{C})$-Knapsack collection contains an instance (*U*, *w*, *v*, *W*, *V*) for which there exists $W'\in \{0,1,\ldots ,W\}$ such that $V_{W'}\ge W'+k$. This instance is a $(\mathcal{H},c)$-Knapsack instance for some $c\in \mathcal{C}$. So, by Lemma 4.23, $(\mathcal{P}, R,h,k)$ is, in particular, a yes-instance of Disk Repacking problem

**Proof of** **Theorem** 1.2:**Putting It All Together.** We are now ready to make the final step of the proof of Theorem 1.2.

The algorithm works as follows. Given an instance $(\mathcal{P}, R,h,k)$ of Disk Repacking problem, it calls the algorithm in Lemma 4.5 to either correctly determine that $(\mathcal{P}, R,h,k)$ is a yes-instance or correctly determine that $(\mathcal{P}, R,h,k)$ is dense and obtain a hole cover $\mathcal H$ of size smaller than *k*. In the first case, the algorithm is done. In the second case, the algorithm proceeds as follows. First, it calls the algorithm in Proposition 4.26 to obtain a $(\mathcal{P},q(h+k))$-universal set $\mathcal C$. Then, it calls the algorithm in Corollary 4.28 to obtain the $(\mathcal{H},\mathcal{C})$-Knapsack collection. Afterwards, it uses the algorithm of Proposition 4.22 to determine whether the $(\mathcal{H},\mathcal{C})$-Knapsack collection contains an instance (*U*, *w*, *v*, *W*, *V*) for which there exists $W'\in \{0,1,\ldots ,W\}$ such that $V_{W'}\ge W'+k$.

The correctness of the algorithm follows from Lemma 4.29. The runtime bound of $(h+k)^{\mathcal {O}(h+k)}\cdot |I|^{\mathcal {O}(1)}$ follows from the runtimes bounds of the algorithms that the algorithm calls, stated in Lemma 4.5, Proposition 4.26, Corollary 4.28, and Proposition 4.22.

This concludes the proof of Theorem 1.2.

## An FPT Approximation for Maximum Disk Repacking

In this section, we use Theorem 1.2 to show that the optimization variant of Disk Repacking problem, called Max Disk Repacking problem, admits an FPT-AS (Fixed-Parameter Tractable Approximation Scheme), when parameterized by *h*. Let us remind that in Max Disk Repacking problem, we are given a packing $\mathcal {P}$ of *n* disks in a rectangle *R* and an integer *h*, and the task is to maximize the number of new disks that can be added to the packing if we are allowed to relocate at most *h* disks of $\mathcal {P}$. Given an instance of Max Disk Repacking problem, we use $\textsf{OPT}_h$ the maximum number of disks that can be added to the input packing if we can relocate at most *h* disks.

We first need an algorithm for the special case of Max Disk Repacking problem, that is, for the optimization version of Disk Appending problem. Let $\textsf{OPT}$ be the maximum number of disks that can be added in a rectangle to complement a given packing $\mathcal {P}$. For a fixed $\varepsilon > 0$, we design an algorithm that returns a packing $\mathcal {P}^*\supseteq \mathcal {P}$ of at least $n + (1-\varepsilon ) \cdot \textsf{OPT}_0$ disks. The algorithm is based on the shifting technique, originally introduced by Hochbaum and Maass [21] (also related to Baker’s technique [4]).

### Lemma 5.1

For any $0< \varepsilon < 1$, there exists an algorithm that for a packing of *n* disks in a rectangle, returns a packing with at least $n + (1-\varepsilon ) \cdot \textsf{OPT}_0$ disks in time $\big (\frac{1}{\varepsilon }\big )^{\mathcal {O}(1/\varepsilon ^2)} \cdot |I|^{\mathcal {O}(1)}$, where |*I*| is the input size.

### Proof

For the scope of this proof, we denote $\textsf{OPT}:=\textsf{OPT}_0$. Let $\mathcal {S}^*$, $|\mathcal {S}^*|=\textsf{OPT}$, be the set of newly added disks in an optimal solution. Let $\ell \ge 1$ be a fixed positive integer. Recall that the instance is contained inside a bounding rectangle *R*. Let us assume that the bottom-left corner of *R* has Cartesian coordinates (0, 0). For every $1 \le i, j \le 2\ell$, let $G_{i, j}$ be a grid of side-length $\ell \times \ell$, with origin at $(-i, -j)$. We first prove the following simple observation.

### Observation 5.2

There exists a pair (*i*, *j*) with $1 \le i, j \le 2\ell$, such that the number of disks of $\mathcal {S}^*$ that do not intersect with the boundary of the grid cells in $G_{i, j}$ is at least $(1-\frac{1}{\ell })^2 \cdot \textsf{OPT}$.

### Proof

Since the diameter of every disk in $\mathcal {S}^*$ is 2, there exists an index $1 \le i \le 2\ell$ such that at most $\frac{1}{\ell }$ fraction of disks from $\mathcal {S}^*$, i.e., at most $\textsf{OPT}/\ell$ disks, intersect the vertical lines $x = a \ell + i$ for integers *a*. Fix this value of index *i*, and let $\mathcal {S}^*_i \subseteq \mathcal {S}^*$ denote the subset of disks that *do not* intersect the vertical lines $x = a \ell + i$. By previous argument, $|\mathcal {S}^*_i| \ge (1-\frac{1}{\ell }) \cdot \textsf{OPT})$. Again, by a similar argument, there exists an index $1 \le j \le 2\ell$, such that at most $\frac{1}{\ell }$ fraction of the disks of $\mathcal {S}^*_i$, intersect the horizontal lines $b \ell + j$ for integers *b*. Fix this integer *j*, and let $\mathcal {S}^*_{i,j} \subseteq \mathcal {S}^*_i$ denote the subset of disks that do not intersect vertical lines $x = a\ell +i$ as well as do not intersect horizontal lines $y = b\ell +j$. It follows that $|\mathcal {S}^*_{i,j}| \ge (1-\frac{1}{\ell }) \cdot |\mathcal {S}^*_i| \ge (1-\frac{1}{\ell })^2 \cdot \textsf{OPT}$. $\square$

For any $1 \le i, j \le n$, and a grid cell *C* in $G_{i, j}$, let $\Pi (C)$ be the following subproblem. Let $\mathcal {P}(C) \subseteq \mathcal {P}$ denote the packing of the original disks that are completely contained in *C*, or partially intersect with *C*. The goal is to add the maximum number of new disks to obtain a packing $\mathcal {P}^*(C)$. Note that the number of original disks in $\mathcal {P}$, as well as the new disks that can be added inside *C*, is upper bounded by $\ell ^2$, which is a constant. Therefore, an optimal solution to $\Pi (C)$ can be found by solving a system of polynomial equations. Let $OPT_{i, j}$ denote the sum of the optimal values for the subproblems $\Pi (C)$, over all grid cells *C* in $G_{i, j}$.

Let $\mathcal {P}(C)$ denote the packing of the original disks that are completely contained in the cell *C*, or partially intersect with *C*. Recall that *C* is a square of size $\ell \times \ell$, and since $\mathcal {P}(C)$ is a packing, $|\mathcal {P}(C)| = \mathcal {O}(\ell ^2)$. Furthermore, the number of new disks that can be added to *C* to obtain a new packing is also upper bounded by $p = \mathcal {O}(\ell ^2)$. We first “guess” the number of new disks, by trying all possible values *q* between 1 and $p = \mathcal {O}(\ell ^2)$. Now, we construct a system of polynomial equations with 2*q* variables and $q(|\mathcal {P}|+4)$ equations, as follows. For every new disk $D_i$ for $1 \le i \le q$, we have two variables corresponding to the *x* and *y* coordinates of its center in the new packing. For every new disk $D_i$, we also add 4 linear equations that restrict the center to lie at a horizontal/vertical distance of at least 1 from the perimeter of the cell, so that the disk $D_i$ lies completely within the cell *C*. Finally, for every disk $D'_j$ in the original packing $\mathcal {P}$, we have an equation that enforces that the distance between the center of $D_i$ and that of $D'_j$ must be at least 2. Now, we solve this system of $\mathcal {O}(\ell ^2)$ variables and $\mathcal {O}(\ell ^4)$ equations in time $\mathcal {O}(\ell )^{\mathcal {O}(\ell ^2)}$ time, using Proposition 2.1.

By Observation 5.2, there exists a pair (*i*, *j*) with $1 \le i,j \le \ell$, such that $\textsf{OPT}_{i, j} \ge \left( 1 - \frac{1}{\ell }\right) ^2 \cdot \textsf{OPT}$, since $\mathcal {S}^*_{i,j}$ as defined in the proof of Observation 5.2 is a feasible solution for the corresponding subproblem. Therefore, for every $1 \le i, j \le 2\ell$, and for every grid cell *C* in $G_{i, j}$, we solve the subproblem $\Pi (C)$, and return the best solution. Note that if we are looking for an $(1-\varepsilon )$-approximation to the number of newly added disks, then $(1-\varepsilon ) \le \left( 1-\frac{1}{\ell }\right) ^2 \le 1- \frac{1}{\ell }$ That is, $\ell = 1/\varepsilon$. Thus, the running time of this algorithm is $\big (\frac{1}{\varepsilon }\big )^{\mathcal {O}(1/\varepsilon ^2)} \cdot |I|^{\mathcal {O}(1)}$. $\square$

Now we design our FPT-AS for Max Disk Repacking problem a combination of Lemma 5.1 and Theorem 1.2. This result is formally (re)stated in the following theorem.

### Theorem 5.3

For any $0< \varepsilon < 1$, there exists an algorithm that, given an instance $(\mathcal {P},R,h)$ of Max Disk Repacking problem, returns a packing $\mathcal {P}^*$ into *R* with at least $n + (1-\varepsilon ) \cdot \textsf{OPT}_h$ disks in time $\max \left\{ \left( \frac{h+1}{\varepsilon }\right) ^{\mathcal {O}(h/\varepsilon )}, \left( \frac{1}{\varepsilon }\right) ^{\mathcal {O}(1/\varepsilon ^2)}\right\} \cdot |I|^{\mathcal {O}(1)} \le \big (\frac{h+1}{\varepsilon }\big )^{\mathcal {O}(h/\varepsilon +1/\varepsilon ^2)} \cdot |I|^{\mathcal {O}(1)}$, where $\textsf{OPT}_h$ is the maximum number of disks that can be added to the input packing if we can relocate at most *h* disks.

### Proof

Let $0<\varepsilon <1$. Consider an instance $(\mathcal {P},R,h)$ of Max Disk Repacking problem. We find the maximum nonnegative integer $k\le 10\,h/\varepsilon$ such that $(\mathcal {P},R,h,k)$ is a yes-instance of Disk Repacking problem the algorithm from Theorem 1.2. This can be done in $\big (\frac{h+1}{\varepsilon }\big )^{\mathcal {O}(h/\varepsilon )} \cdot |I|^{\mathcal {O}(1)}$ time. Next, we run the algorithm from Lemma 5.1 for (*G*, *R*) for $\varepsilon '=\frac{1}{2}\varepsilon$, i.e., assuming that relocations of disks are not allowed. The algorithm runs in $\big (\frac{1}{\varepsilon }\big )^{\mathcal {O}(1/\varepsilon ^2)} \cdot |I|^{\mathcal {O}(1)}$ time and returns a solution of size $k'$. We set $k^*=\max \{k,k'\}$. We claim that $(1-\varepsilon )\textsf{OPT}_h\le k^* \le \textsf{OPT}_h$. The second inequality is trivial. To show that $(1-\varepsilon )\textsf{OPT}_h\le k^*$, we consider two cases.

Suppose that $\textsf{OPT}_h\le 10h/\varepsilon$. Then $\textsf{OPT}_h=k$ as the algorithm from Theorem 1.2 is exact and $(1-\varepsilon )\textsf{OPT}_h\le \textsf{OPT}_h=k\le k^*$.

Assume that $\textsf{OPT}_h> 10h/\varepsilon$. Let $\mathcal {S}$ be the set of added disks in an optimum solution for $(\mathcal {P},R,h)$ and let $\mathcal {L}\subseteq \mathcal {P}$ be the set of relocated disks. Denote by $\textsf{OPT}'$ the maximum number of disks that can be added to $\mathcal {P}$ without relocations. Observe that every disk in $\mathcal {L}$ intersects at most 5 disks of $\mathcal {S}$. Therefore, $\textsf{OPT}'\ge |\mathcal {S}|-5|\mathcal {L}|\ge \textsf{OPT}_h-5h$. By Lemma 5.1, $(1-\varepsilon /2)\textsf{OPT}'\le k'$. We obtain that $(1-\varepsilon /2)(\textsf{OPT}_h-5h)\le k'\le k^*$. Because $\textsf{OPT}_h> 10\,h/\varepsilon$, $k^*\ge (1-\varepsilon /2)(\textsf{OPT}_h-\varepsilon \textsf{OPT}_h/2)=(1-\varepsilon /2)^2\textsf{OPT}_h\ge (1-\varepsilon )\textsf{OPT}_h$. This proves the claim.

We conclude that $k^*$ is the required approximation of $\textsf{OPT}_h$. To conclude the proof, note that the algorithms from Theorem 1.2 and Lemma 5.1 can be adapted to return solutions, that is, the sets of added and relocated disks. $\square$

## Conclusion and Open Questions

We have shown in Theorem 1.1 that Disk Repacking problem is ${{\,\textrm{NP}\,}}$-hard even if $h=0$. On the other hand, by Theorem 1.2, Disk Repacking problem when parameterized by *k* and *h*. Both theorems naturally lead to the question about parameterization by *k* only. The difficulty here is that even for adding one disk, one has to relocate many disks. Already for $k=1$, we do not know, whether the problem is in ${{\,\textrm{P}\,}}$  or is ${{\,\textrm{NP}\,}}$-hard.

Another natural question stemming from Theorem 1.2 is about kernelization of Disk Repacking problem. Does Disk Repacking problem a polynomial kernel with parameters *k* and *h*? (We refer to books [11, 16] for an introduction to kernelization).

Finally, approximation of Disk Repacking problem an interesting research direction. In Theorem 1.3 we demonstrated that our ${{\,\textrm{FPT}\,}}$ algorithm can be used to construct an ${{\,\mathrm{FPT-AS}\,}}$ with respect to *h* for Max Disk Repacking problem. We leave open the question about polynomial approximation. Another open question concerns the approximability of the minimum number of relocations *h* for a given *k*. Already for $k=1$ finding a good approximation of *h* is a challenging problem.

## Data Availability

There is no data associated with the manuscript.

## Footnotes

1 In the literature, it is often required for geometric packings that a packing should be maximal. In particular, for disk packing, every disk should touch either the bounding rectangle or another disk. However, in our problem, the task is to add a specified number of new disks to a given family and this makes the maximality condition in our case very artificial.

2 Placing a disk with it center at *O* corresponds to selecting the vertex to be included in an independent set.

3 Note that $(\mathcal{P}{\setminus }\mathcal{P}^*) \cup (\mathcal{P}^*{\setminus }\mathcal{P})$ are essentially the (unit) disks that are moved by the solution plus the disks that are newly added by the solution.

4 Whether a region contains a specific disk or not can be directly tested by the definition of its description.
